# Generalizing β-VDR-based derivative computation for robust source edge detection and depth estimation from potential field data

**DOI:** 10.1038/s41598-026-36635-7

**Published:** 2026-01-18

**Authors:** Sesan Cornelius Falade, Ayomiposi Henry Falade

**Affiliations:** 1 Department of Physical Sciences, Landmark University, Omu-aran, Nigeria; 2 Department of Geology, Obafemi Awolowo University, Ile-Ife, Nigeria

**Keywords:** Robust derivatives, Source edge detection, Depth estimation, Chad basin, Potential field method, Engineering, Materials science, Mathematics and computing

## Abstract

The derivatives of potential field data are essential for source edge detection and depth estimation. A recently introduced vertical derivative method, named β-VDR, has received remarkable recognition in the literature due to its robust vertical derivative approximation. In this study, we compacted the β-VDR formula and extended the method to horizontal derivative calculation to reduce its theoretical computational cost and improve its robustness for edge detection and depth estimation from profile and gridded potential field data. We demonstrated the superior performance of our generalized method through 2D and 3D synthetic tests and showed its applicability on real data.

## Introduction

The interpretation of potential field data from gravity or magnetic surveys is a fundamental process in geophysical exploration and crustal studies for mapping and characterizing subsurface geological structures non-invasively^1,2^. The key targets in these surveys are features such as geological contacts, faults, and dykes, whose positions and geometries must be known for successful resource exploration and structural analysis. Derivative-based methods are used to detect these structural features and estimate their depths. However, the effectiveness of these methods is often limited by the noise sensitivity of the operators required for derivative computation.

Potential field measurements are often contaminated by noise, which can originate from instrumental errors, cultural features, or near-surface geological disturbances. The presence of noise in the measurements, even at low levels, can severely distort the computed derivatives, often obscuring the underlying, meaningful geological signal, leading to inaccurate edge detection and unreliable depth estimates.

The ill-conditioned problem arises from the intrinsic high-frequency filtering property of the derivative operators, which disproportionately amplifies high-frequency noise in the measured data. This renders conventional derivative operators ineffective for robust interpretation in noisy environments and thus poses a significant challenge in potential field interpretation.

Efforts have been made in the literature to address the instability of derivative operators for potential field data interpretation (e.g.,^3–7^). The vertical and horizontal derivatives used for source edge detection and depth estimation are often computed in the Fourier domain^8^, whose derivative operators are notoriously ill-conditioned in the presence of noise, as will be demonstrated in this study. Fedi & Florio^9^ introduced the Integrated Second Vertical Derivative (ISVD) method, which computes the first vertical derivative by first computing the vertical integral of the potential data in the Fourier domain, then the second-order horizontal derivative using finite difference (FD) approximations and the Laplace Equation. This method performs better than the Fourier-based vertical derivative but is only effective at low noise levels. There have been other attempts to improve vertical derivatives by combining FD and upward continuation^7,10,11^.

Oliveira & Pham^12^ proposed a more robust vertical derivative method, named $\:\beta\:$-VDR, using a finite-difference formula based on upward continuation. They demonstrated the superior performance of the $\:\beta\:$-VDR method over the backward difference method of Florio et al.^10^ and the higher-order vertical derivative formula of Tran & Nguyen^7^, among other methods, using synthetic models. This method, although recent, has received remarkable recognition by several authors. Vertical derivatives computed using the $\:\beta\:$-VDR method have been used to improve Euler Deconvolution^13,14^, Tilt-depth^15^, downward continuation^16^, Elliott Function^17^, total horizontal gradient^18^, among others (e.g.,^19^). These applications show the increasing importance of the $\:\beta\:$-VDR method in modern potential-field interpretation.

Despite its strengths, the β-VDR method has notable limitations. The vertical derivative of the $\:\beta\:$-VDR method was proposed to be used with FD horizontal derivatives for edge detection and depth estimation^12^. This creates an unbalanced enhancement since the $\:\beta\:$-VDR has greater robustness than FD, as will be demonstrated in this study. This discrepancy often results in asymmetric noise suppression, which can introduce inconsistencies in source-edge detection and depth estimation when using edge-detectors that critically depend on balanced vertical and horizontal derivatives. Therefore, there is a need for the formulation of an equivalent β-VDR’s horizontal derivative operator to achieve balanced and stable edge enhancement.

Another significant limitation of the $\:\beta\:$-VDR method is its high computational cost compared to standard vertical derivative operators. The procedure demands five upward continuations, which require five forward and inverse Fourier transforms. Even with the advent of fast Fourier transform algorithms, this can become computationally expensive for large datasets, such as high-resolution aeromagnetic surveys or regional grids with millions of data points, and may limit the method’s applicability in operational or real-time processing environments. Reducing the number of required Fourier transform operations can improve the computational speed.

To address these limitations and improve source edge enhancement and depth estimation from potential field data, we generalized the $\:\beta\:$-VDR method by first compacting its formula using Fourier-space domain relationships to produce an equivalent frequency domain operator requiring only one forward and inverse Fourier transform operation, instead of five, for the entire process. Then, we extended the method to horizontal derivatives in 1D and 2D. Furthermore, we demonstrated the effectiveness of the proposed method using 2D and 3D potential field models and real-life aeromagnetic data.

## Methods

The $\:\beta\:$-VDR vertical derivative of potential field anomaly, $\:f$, is defined as^12^:

1 $$\:\frac{\partial f}{\partial z}=\frac{{a}_{1}f\left({h}_{1}\right)+{a}_{2}f\left({h}_{2}\right)+{a}_{3}f\left({h}_{3}\right)+{a}_{4}f\left({h}_{4}\right)+{a}_{5}f\left({h}_{5}\right)}{\varDelta z}$$

where coefficients $\:{a}_{1},\dots\:,{a}_{5}$ are given as

2 $$\begin{aligned}\:{a}_{1}&=(2{\beta\:}^{3}+15{\beta\:}^{2}+35\beta\:+25)/12,\:\\{a}_{2}&=(-8{\beta\:}^{3}-54{\beta\:}^{2}-104\beta\:-48)/12,\:\\ {a}_{3}&=(12{\beta\:}^{3}+72{\beta\:}^{2}+114\beta\:+36)/12,\\{a}_{4}&=(-8{\beta\:}^{3}-42{\beta\:}^{2}-56\beta\:-16)/12,\:\\{a}_{5}&=(2{\beta\:}^{3}+9{\beta\:}^{2}+11\beta\:+3)/12,\end{aligned}$$

and $\:f\left({h}_{j}\right)$ are the potential field anomaly $\:f$ upward continued to heights

3 $$\:{h}_{j}=\beta\:\varDelta z+\left(j-1\right)\varDelta z,\:\:\:\:\:\:j=1,\dots\:,\:5$$

above the observation plane, $\:\varDelta z$ is a fraction of the profile data point spacing or grid spacing that determines the vertical increment of the upward continuation levels, and the positive constant $\:\beta\:$ is a user-defined stabilizing parameter. The $\:\beta\:$ parameter controls the level of robustness of the method; $\:\beta\:=0$ produces derivatives similar to conventional derivatives. Oliveira and Pham^12^ showed that a vertical increment $\:\varDelta z$ of one tenth of the data point spacing and a stabilizing parameter ranging from 40 to 60 give a balance between accuracy and low sensitivity to noise.

In the frequency domain, the upward continuation of potential field anomaly $\:f$ to a given height $\:{h}_{j}$ above the observation plane is defined as^8^:

4 $$\:\mathcal{F}\left[f\left({h}_{j}\right)\right]=\:{e}^{-{h}_{j}\left|k\right|}\mathcal{F}\left[f\right]$$

where $\:\mathcal{F}$ denotes the Fourier transform and $\:\left|k\right|$ is the magnitude of direction wavenumber(s). Thus,

5 $$\:f\left({h}_{j}\right)={\mathcal{F}}^{-1}\left[\:{e}^{-{h}_{j}\left|k\right|}\mathcal{F}\left[f\right]\:\right],\:\:\:\:\:j=1,\dots\:,\:5$$

This implies that the computation of β-VDR vertical derivative requires five upward continuation operations, demanding five pairs of forward and inverse Fourier transforms of the potential field data. Efficient implementation of Eq. 5 will require at least one forward Fourier transform and five inverse Fourier transform operations.

To reduce the computational cost of the β-VDR method, we redefined Eq. 1 as follows:

6 $$\:\frac{\partial f}{\partial z}=\sum_{j=1}^{5}{b}_{j}f\left({h}_{j}\right)$$

where $\:{b}_{j}$ are arbitrary constants defined as

7 $$\:{b}_{j}=\frac{{a}_{j}}{\varDelta z}\:\:\:\:\:\:\:j=1,\dots\:,\:5$$

The Fourier transform of Eq. 6 can be expressed as

8 $$\:\mathcal{F}\left[\frac{\partial f}{\partial z}\right]=\mathcal{F}\left[\:\sum_{j=1}^{5}{b}_{j}f\left({h}_{j}\right)\:\right]$$

One of the properties of the Fourier transform is that it is a linear operation^8^. The multiplication of a function by an arbitrary constant in the space domain is equivalent to scaling its Fourier spectrum by the same constant in the frequency domain. That is,

9 $$\:{b}_{j}f\left({h}_{j}\right)\leftrightarrow\:{b}_{j}\mathcal{F}\left[f\left({h}_{j}\right)\right]$$

Also, addition in the space domain is equivalent to addition in the frequency domain. Thus,

10 $$\:\sum_{j=1}^{5}{b}_{j}f\left({h}_{j}\right)\leftrightarrow\:\sum_{j=1}^{5}{b}_{j}\mathcal{F}\left[f\left({h}_{j}\right)\right]$$

or

11 $$\:\mathcal{F}\left[\sum_{j=1}^{5}{b}_{j}f\left({h}_{j}\right)\right]=\sum_{j=1}^{5}{b}_{j}\mathcal{F}\left[f\left({h}_{j}\right)\right]$$

Equation 8, therefore, becomes

12 $$\:\mathcal{F}\left[\frac{\partial f}{\partial z}\right]=\sum_{j=1}^{5}{b}_{j}\mathcal{F}\left[f\left({h}_{j}\right)\right]$$

Substituting Eq. 4 into 12, we have

13 $$\:\mathcal{F}\left[\frac{\partial f}{\partial z}\right]=\left(\sum_{j=1}^{5}{b}_{j}\:{e}^{-{h}_{j}\left|k\right|}\right)\mathcal{F}\left[f\right]$$

The inverse transform gives

14 $$\:\frac{\partial f}{\partial z}={\mathcal{F}}^{-1}\left[\left(\sum_{j=1}^{5}{b}_{j}\:{e}^{-{h}_{j}\left|k\right|}\right)\mathcal{F}\left[f\right]\right]$$

which is a compact form of Eq. 1 requiring only one forward and inverse Fourier transform operation.

To extend the $\:\beta\:$-VDR method for the computation of horizontal derivative, we used the 2D Laplace Equation to obtain:

15 $$\:\frac{{\partial}^{2}f}{\partial{x}^{2}}=-\frac{{\partial}^{2}f}{\partial{z}^{2}}$$

Taking the Fourier transform of both sides yields

16 $$\:\left(ik\right)\mathcal{F}\left[\frac{\partial f}{\partial x}\right]=-{\psi}_{z}^{2}\mathcal{\:}\mathcal{F}\left[f\right]$$

where $\:i$ is the imaginary unit, $\:k$ is the wavenumber, and $\:{\psi}_{z}$ is the vertical derivative response defined as:

17 $$\:{\psi}_{z}=\left\{\begin{array}{c}\sum_{j=1}^{5}{b}_{j}\:{e}^{-{h}_{j}\left|k\right|}\:\:\:\:\:\:\:\:\:\:\:\:\:\:\:(\beta\:\mathrm{-VDR})\\\:\\\:\left|k\right|\:\:\:\:\:\:\:\:\:\:\:\:\:\:\:\:\:\:\:\:\:\:\:\:\:\:\:\:\:\:\:\left(standard\right)\end{array}\right.$$

To provide a balance between the standard and β-VDR vertical derivative filters, and to ensure that only the first-order smoothening effect of β-VDR is applied for horizontal derivative computation, we expressed $\:{\psi}_{z}^{2}$ as

18 $$\:{\psi}_{z}^{2}=\left|k\right|\sum_{j=1}^{5}{b}_{j}\:{e}^{-{h}_{j}\left|k\right|}$$

Equation 16 becomes

19 $$\begin{aligned}\:\mathcal{F}\left[\frac{\partial f}{\partial x}\right]&=\left(-\frac{\left|k\right|}{ik}\sum_{j=1}^{5}{b}_{j}\:{e}^{-{h}_{j}\left|k\right|}\right)\mathcal{\:}\mathcal{F}\left[f\right]\\ &=\left(i\:\mathrm{s}\mathrm{g}\mathrm{n}\left(k\right)\sum_{j=1}^{5}{b}_{j}\:{e}^{-{h}_{j}\left|k\right|}\right)\mathcal{\:}\mathcal{F}\left[f\right]\end{aligned}$$

and

20 $$\:\frac{\partial f}{\partial x}={\mathcal{F}}^{-1}\left[\left(i\:\mathrm{s}\mathrm{g}\mathrm{n}\left(k\right)\sum_{j=1}^{5}{b}_{j}\:{e}^{-{h}_{j}\left|k\right|}\right)\mathcal{\:}\mathcal{F}\left[f\right]\right]$$

which can be used to compute the horizontal derivative of profile data.

For gridded data, we considered the relationship between the x-derivative and second vertical derivative in the frequency domain:

21 $$\:\mathcal{F}\left[\frac{\partial f}{\partial x}\right]=\frac{i{k}_{x}}{{\left|k\right|}^{2}}\mathcal{\:}\mathcal{F}\left[\frac{{\partial}^{2}f}{d{z}^{2}}\right]$$

where $\:\left|k\right|=\sqrt{{k}_{x}^{2}+{k}_{y}^{2}}$, and $\:{k}_{x},\:{k}_{y}$ are wavenumbers in x- and y-directions, respectively. Thus,

22 $$\begin{aligned}\mathcal{F}\left[\frac{\partial f}{\partial x}\right]&=\frac{i{k}_{x}}{{\left|k\right|}^{2}}{\psi}_{z}^{2}\mathcal{\:}\mathcal{F}\left[f\right]\\&=\frac{i{k}_{x}}{\left|k\right|}\sum_{j=1}^{5}{b}_{j}\:{e}^{-{h}_{j}\left|k\right|}\mathcal{\:}\mathcal{F}\left[f\right]\end{aligned}$$

and

23 $$\:\frac{\partial f}{\partial x}={\mathcal{F}}^{-1}\left[\left(\frac{i{k}_{x}}{\left|k\right|}\sum_{j=1}^{5}{b}_{j}\:{e}^{-{h}_{j}\left|k\right|}\right)\mathcal{\:}\mathcal{F}\left[f\right]\right]$$

Likewise,

24 $$\:\frac{\partial f}{\partial y}={\mathcal{F}}^{-1}\left[\left(\frac{i{k}_{y}}{\left|k\right|}\sum_{j=1}^{5}{b}_{j}\:{e}^{-{h}_{j}\left|k\right|}\right)\mathcal{\:}\mathcal{F}\left[f\right]\right]$$

Generally,

25 $$\:\frac{{\partial}^{n}f(x,z)}{\partial {z}^{n}}={\mathcal{F}}^{-1}\left[{\left(\sum_{j=1}^{5}{b}_{j}\:{e}^{-{h}_{j}\left|k\right|}\right)}^{n}\mathcal{F}\left[f(x,z)\right]\right]$$

26 $$\:\frac{{\partial}^{n}f(x,z)}{\partial {x}^{n}}={\mathcal{F}}^{-1}\left[{\left(i\:\mathrm{s}\mathrm{g}\mathrm{n}\left(k\right)\sum_{j=1}^{5}{b}_{j}\:{e}^{-{h}_{j}\left|k\right|}\right)}^{n}\mathcal{\:}\mathcal{F}\left[f(x,z)\right]\right]$$

27 $$\:\frac{{\partial}^{n}f(x,y,z)}{\partial {z}^{n}}={\mathcal{F}}^{-1}\left[{\left(\sum_{j=1}^{5}{b}_{j}\:{e}^{-{h}_{j}\left|k\right|}\right)}^{n}\mathcal{F}\left[f(x,y,z)\right]\right]$$

28 $$\:\frac{{\partial}^{n}f(x,y,z)}{\partial {x}^{n}}={\mathcal{F}}^{-1}\left[{\left(\frac{i{k}_{x}}{\left|k\right|}\sum_{j=1}^{5}{b}_{j}\:{e}^{-{h}_{j}\left|k\right|}\right)}^{n}\mathcal{\:}\mathcal{F}\left[f(x,y,z)\right]\right]$$

29 $$\:\frac{{\partial}^{n}f(x,y,z)}{\partial {y}^{n}}={\mathcal{F}}^{-1}\left[{\left(\frac{i{k}_{y}}{\left|k\right|}\sum_{j=1}^{5}{b}_{j}\:{e}^{-{h}_{j}\left|k\right|}\right)}^{n}\mathcal{\:}\mathcal{F}\left[f(x,y,z)\right]\right]$$

From here on, the horizontal derivative operators formulated from $\:\beta\:$-VDR are called $\:\beta\:$-HDR, and a combination of the compact $\:\beta\:$-VDR and $\:\beta\:$-HDR for vertical and horizontal derivative computations is called the $\:\beta\:$-VDR-with-$\:\beta\:$-HDR method.

### Implementation details for profile

The vertical and horizontal derivatives of potential field data may be computed as follows:

- Precompute filter parameters $\:b[1..5]$ and h[1.5] using user-defined stabilization parameter $\:\beta\:$ and vertical increment $\:\varDelta z$. Default values: $\:\beta\:=50$ and $\:\varDelta z=0.1\times\:\:$data point spacing.
- Prepare the data for FFT (e.g., filling no-data areas, removal of trend, padding, and tapering to avoid spectral leaks and artifacts).
- Transform the prepared data to the frequency domain using Fast Fourier Transform (FFT) to obtain the Fourier spectrum $\:F$.
- Initialize $\:G$ as a complex array having the same length as *F.*
- For each Fourier frequency $\:k$.

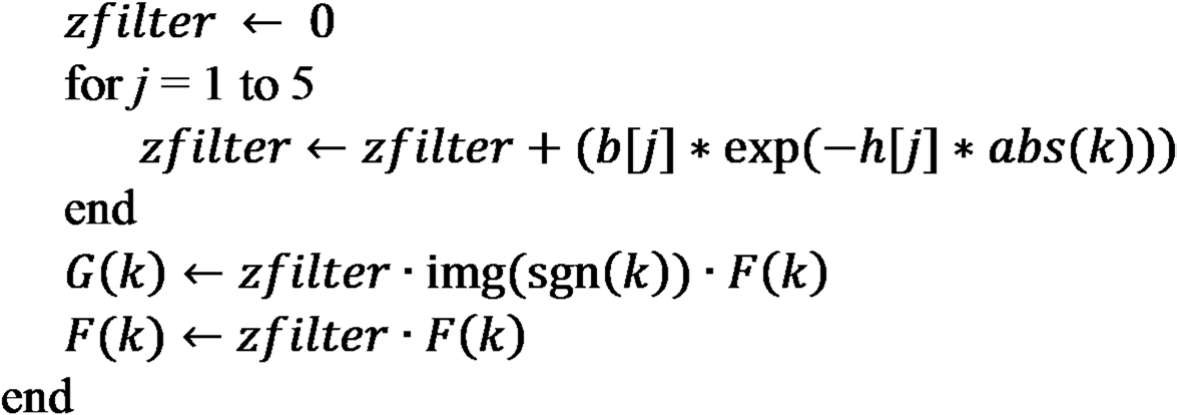

- Compute inverse FFT transform of $\:F$ to obtain vertical derivative.
- Compute inverse FFT transform of $\:G$ to obtain horizontal derivative.

### Implementation details for grid

The vertical and horizontal derivatives of a grid may be computed as follows:

- Precompute filter parameters $\:b[1..5]$ and h[1.5] using user-defined stabilization parameter $\:\beta\:$ and vertical increment $\:\varDelta z$. Default values: $\:\beta\:=50$ and $\:\varDelta z=0.1\times\:\:$grid spacing.
- Prepare the gridded data for FFT (e.g., filling empty grid nodes, removal of trend, padding, and tapering to avoid spectral leaks and artifacts).
- Transform the prepared data into the frequency domain using FFT to obtain the Fourier spectrum $\:F$.
- Initialize $\:G$ as a complex grid having the same size as *F.*
- Initialize $\:H$ as a complex grid having the same size as *F*.
- For each Fourier frequency $\:{k}_{y}$.

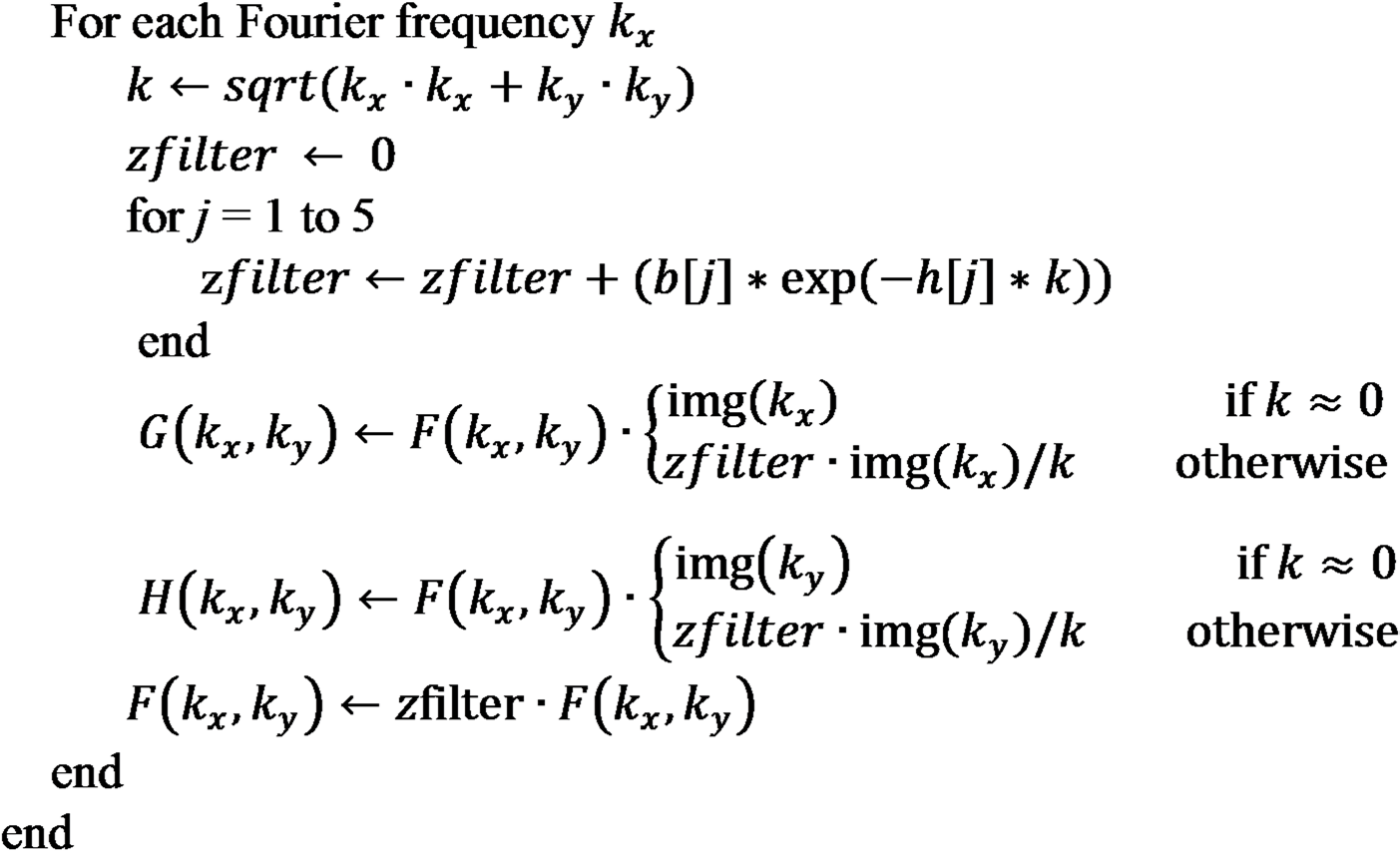

- Compute inverse FFT transform of $\:F$ to obtain vertical derivative.
- Compute inverse FFT transform of $\:G$ to obtain x-derivative.
- Compute inverse FFT transform of $\:H$ to obtain y-derivative.

### Test methods

For our tests, we considered $\:\beta\:$ = 50 and $\:\varDelta z$ as one tenth of the data point spacing, as recommended by Oliveira & Pham^12^ for noisy data. Our data preparation for FFT involved padding the original data by at least 10% to the next power of two and filling the padded regions using the Maximum Entropy Prediction (MEP) technique^20,21^, such that the data is smoothly periodic across opposite edges.

We demonstrated the robustness of the β-VDR-with-β-HDR method for source edge detection and depth estimation using 2D and 3D synthetic examples in comparison with conventional frequency domain derivative filters, ISVD with the FD method, and β-VDR with the FD method. Furthermore, we showed the applicability of our method on real data by interpreting the high-resolution aeromagnetic data over parts of Chad Basin, Nigeria, using derivatives computed with β-VDR-with-β-HDR implementation.

To properly showcase the effectiveness of our derived method, we used the Total Gradient (TG) method for edge detection and depth estimation. The TG method, also known as the Analytic Signal Amplitude method, is one of the most popular source edge enhancement and depth estimation methods. Its high sensitivity to noise^22^ and requirement for both vertical and horizontal derivatives make it a good choice for this study. As the name implies, the TG is the total gradient magnitude of the potential field $\:f$^ 22^:

30 $$\:T{G}_{2D}=\sqrt{{\left(\frac{\partial\:f}{\partial\:x}\right)}^{2}+{\left(\frac{\partial\:f}{\partial\:z}\right)}^{2}}$$

in 2D and

31 $$\:T{G}_{3D}=\sqrt{{\left(\frac{\partial\:f}{\partial\:x}\right)}^{2}+{\left(\frac{\partial\:f}{\partial\:y}\right)}^{2}+{\left(\frac{\partial\:f}{\partial\:z}\right)}^{2}}$$

in 3D. The peaks of the TG indicate the source locations. The source depth is estimated using^22^:

32 $$\:Z=\sqrt{-\frac{2\alpha\:S}{K}}$$

where $\:S$ is the amplitude, and $\:K$ is the curvature (or most negative curvature in grid form) of the TG at the peak location. Parameter $\:\alpha\:=(SI+1)/2$ is a function of the Structural Index (SI) of the source.

## Results and discussion

We assessed the performance of our generalized method, which we refer to as β-VDR-with-β-HDR, for robust edge detection and depth estimation using the Total Gradient (TG) method across 2D and 3D synthetic noisy environments, and in a real-world application over part of the Chad Basin, Nigeria, where geological targets are often obscured by thick sedimentary cover or cultural features.

In this section, we distinguish the TGs used to estimate the source locations and depths by the methods we used to compute their derivatives. We refer to the TGs computed using conventional frequency domain filters as FFT-TG, ISVD with FD horizontal derivative filters as ISVD-TG, the method of Oliveira & Pham^12^ (β-VDR with FD horizontal derivative filters) as β-VDR-TG, and our generalized method as β-VDR-with-β-HDR-TG. The results from both synthetic tests and real data applications are presented and discussed in the following subsections.

### 2D synthetic model tests

**Fig. 1:**
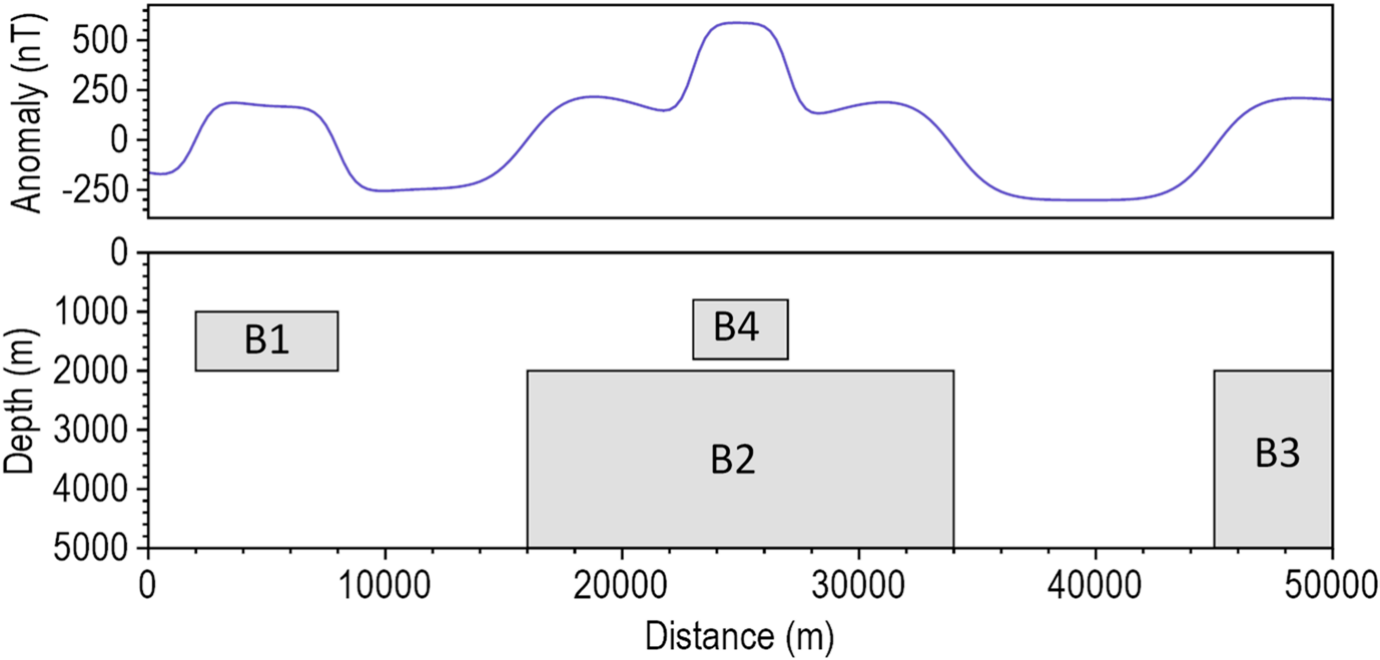
A view of the 2D synthetic model and its magnetic response.

We first considered a model of four 2D magnetic source bodies whose geometric parameters are presented in Table 1. Each body has equal susceptibility (0.005 in CGS units). The inducing field has an intensity of 50,000 nT, with an inclination of $\:90^\circ\:$ and a declination of $\:0^\circ\:$. Figure 1 shows the model source bodies and their corresponding magnetic response.

**Table 1:** Geometric parameters of four 2D magnetic bodies.

Block	Top (m)	Bottom (m)	Left (m)	Right (m)
B1	1000	2000	2000	8000
B2	2000	5000	16,000	34,000
B3	2000	5000	45,000	80,000
B4	800	1800	23,000	27,000

**Fig. 2:**
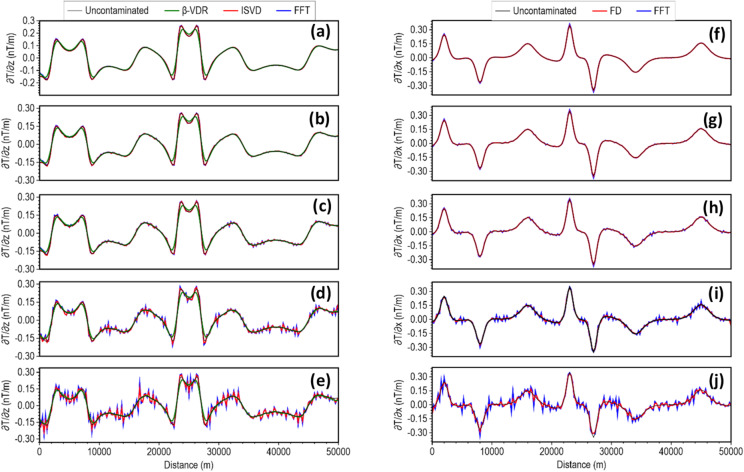
Vertical derivatives (**a**–**e**) and horizontal derivatives (**f**–**j**) of the magnetic model response contaminated with Gaussian noise. The noise has standard deviations of 0.1 nT (**a**,**f**), 0.5 nT (**b**,**g**), 1 nT (**c**,**h**), 3 nT (**d**,**i**), and 5 nT (**e**,**j**).

Figure 2a–e clearly illustrate the superior stability of the β-VDR (green line) compared to the ISVD (red line) and the standard FFT filter (blue line). The magnetic anomaly of the model was contaminated with random Gaussian noise having standard deviations of 0.1, 0.5, 1.0, 3.0 and 5 nT. These standard deviations correspond to approximately 0.02%, 0.1%, 0.2%, 0.5%, and 1.0% of the maximum anomaly amplitude, respectively. The vertical derivative of the uncontaminated anomaly is represented in black. The results (Fig. 2) showed that ISVD offers better stability than FFT. Its response, however, degraded significantly and became highly oscillatory as the noise level increased beyond 1 nT. In contrast, the β-VDR filter maintained a remarkably smooth and coherent derivative signal, even at the highest noise level (5 nT).

The sensitivities of the two most common horizontal derivative methods, FFT (blue line) and FD (red line), to noise are illustrated in Fig. 2e–j at the different noise levels considered. As expected, the FD filter provided better stability than the FFT filter, but became unstable as the noise level increased. A comparison of Fig. 2e with Fig. 2j shows that β-VDR is significantly more stable than the FD. Combining the β-VDR vertical derivative with the FD horizontal derivative for edge detection and depth estimation reduces the robustness of the β-VDR method. Thus, the generalization of the β-VDR method through the introduction of an equivalent horizontal derivative, β-HDR, is fundamentally necessary for robust source edge detection and depth estimation to maintain stability across all derivative components.

**Fig. 3:**
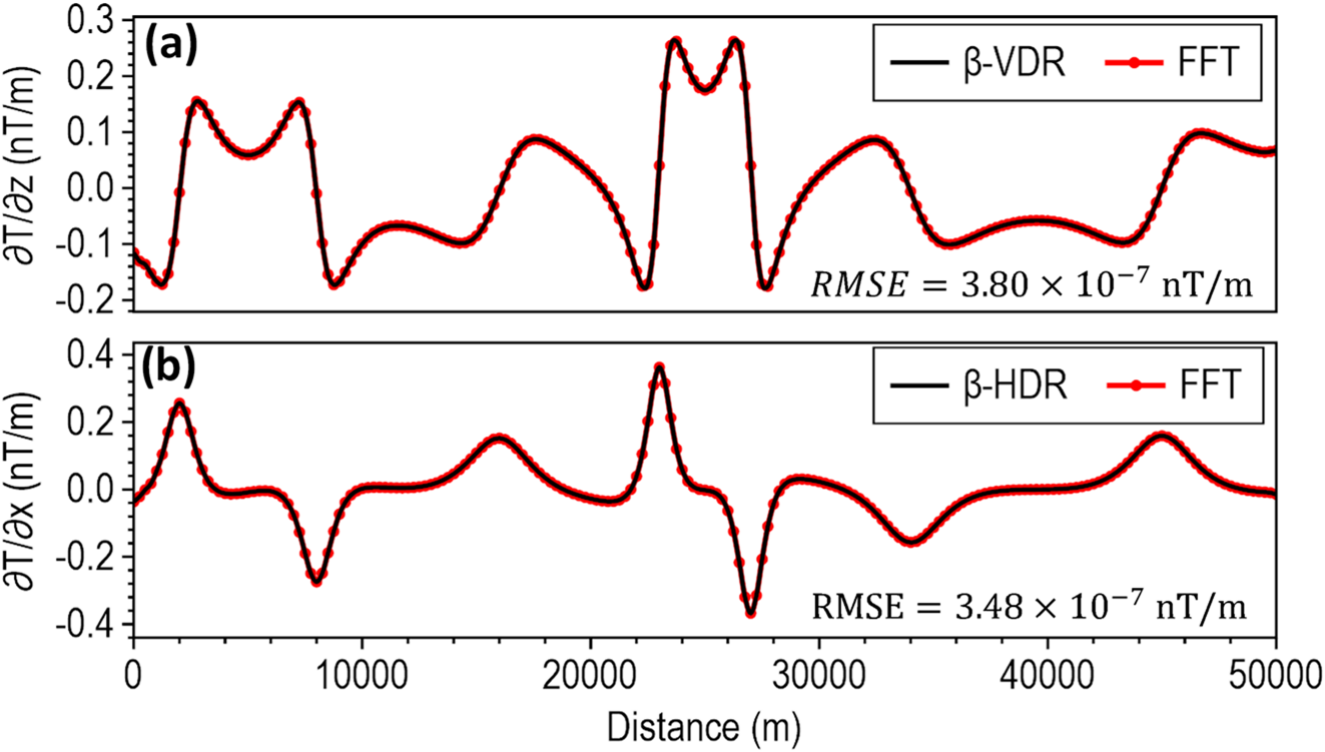
(**a**) Vertical and (**b**) horizontal derivatives computed with FFT derivative filters (red circles) and our derived formula (black line).

**Fig. 4:**
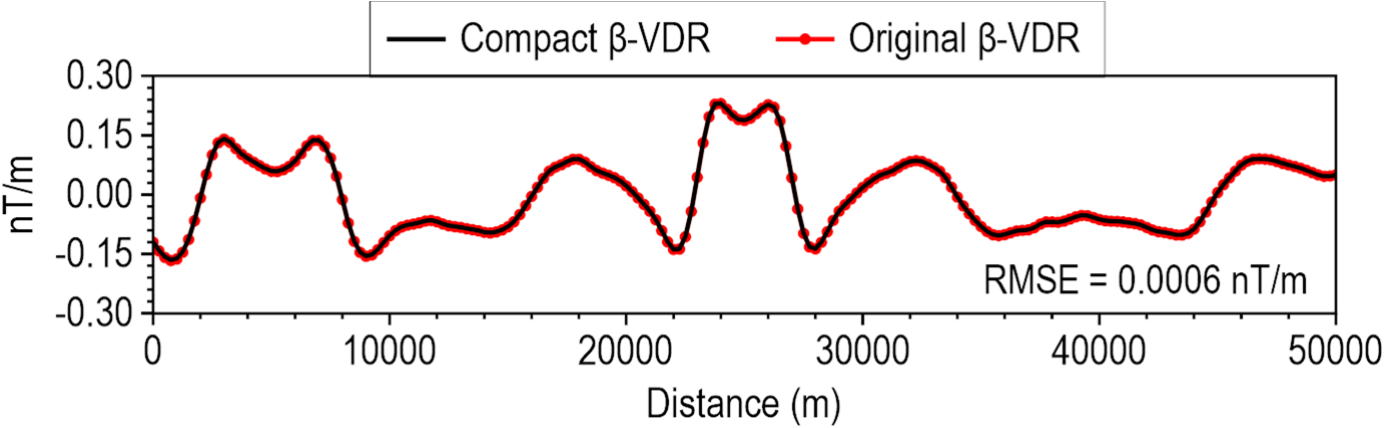
Comparison of β-VDR vertical derivatives computed using the original method^12^ (red circles) and using our compact method (black line).

To demonstrate the theoretical equivalence of the β-VDR-with-β-HDR method to the standard FFT method, we compared their vertical and horizontal derivatives without stabilizing the β-VDR-with-β-HDR (i.e., using $\:\beta\:=0$) on the synthetic model’s uncontaminated magnetic anomaly. This yielded a remarkably low Root Mean Square Error (RMSE) of $\:3.80\times\:{10}^{-7}\:\mathrm{n}\mathrm{T}/\mathrm{m}$ for vertical derivative and $\:3.48\times\:{10}^{-7}\:\mathrm{n}\mathrm{T}/\mathrm{m}$ for horizontal derivative (Fig. 3); thereby verifying the mathematical correctness and consistency of our derived compact β-VDR and formulated β-HDR. We further compared the compact form of β-VDR with its original form^12^ using $\:\beta\:=50$ at 5 nT noise level. This also yielded a low RMSE of 0.0006 nT/m (Fig. 4), demonstrating close agreement with the original. The slight non-zero RMSE value is attributed to the difference in their numerical FFT operations-the original form requires five sets of forward and inverse transforms, while the compact form requires only one, offering significant computational efficiency and makes the compact form less prone to errors introduced by tapering and FFT numerical approximations.

**Fig. 5:**
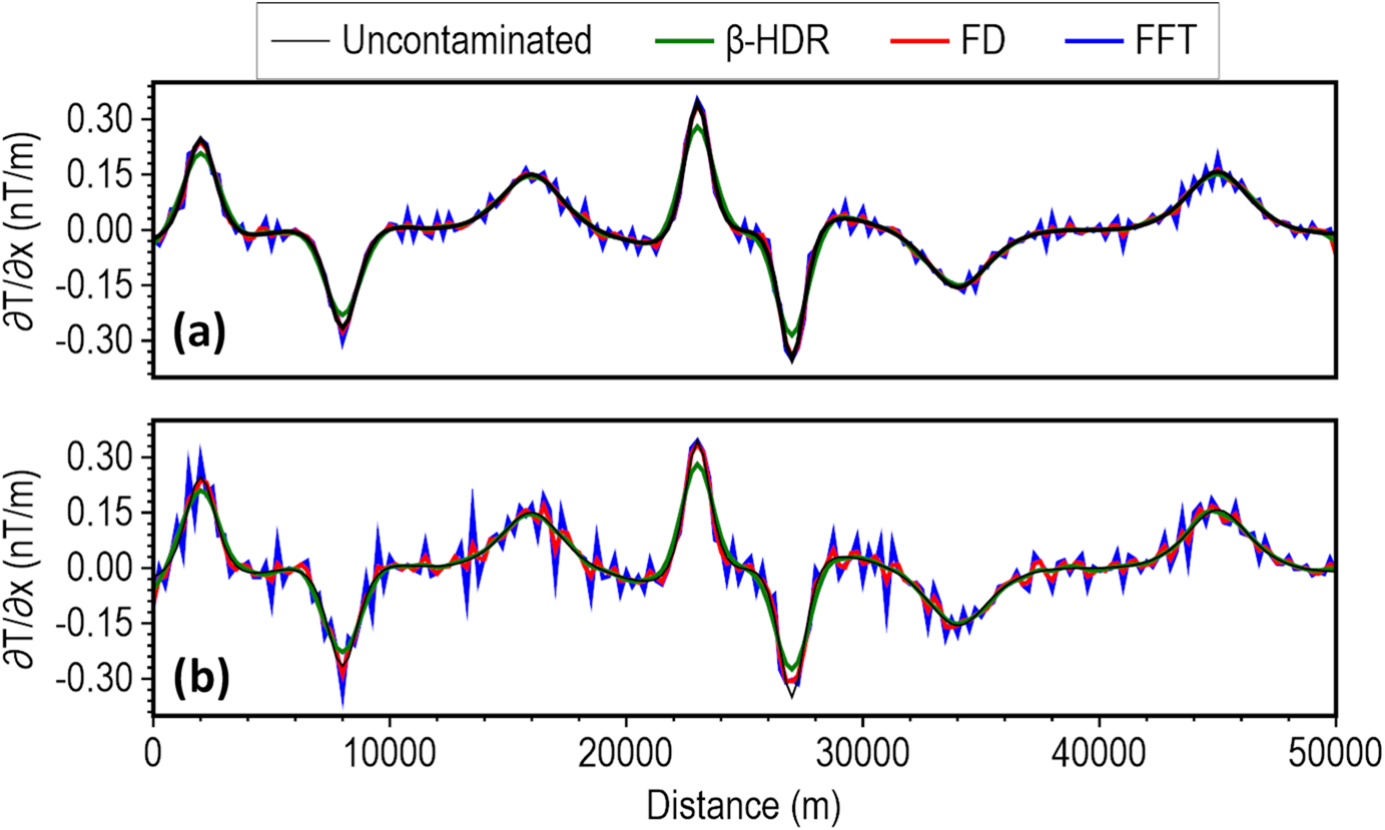
Horizontal derivatives of β-HDR (green line) compared with FD (red line) and FFT (blue line) for the magnetic model response with Gaussian noise having standard deviations of (**a**) 3 nT and (**b**) 5 nT. The black line represents the horizontal derivative of the uncontaminated data.

To demonstrate the stability of the β-HDR in comparison with the other horizontal derivative computation methods (FFT and FD), we computed the horizontal derivatives at noise levels of 3 and 5 nT, and obtained results showing that the β-HDR has greater stability (Fig. 5). Unlike the standard FFT filter, which is highly sensitive to noise, and FD, which becomes unstable at these noise levels, β-HDR maintained a consistent and smooth response, thereby ensuring reliable edge detection and depth estimation under these high noise conditions.

**Fig. 6:**
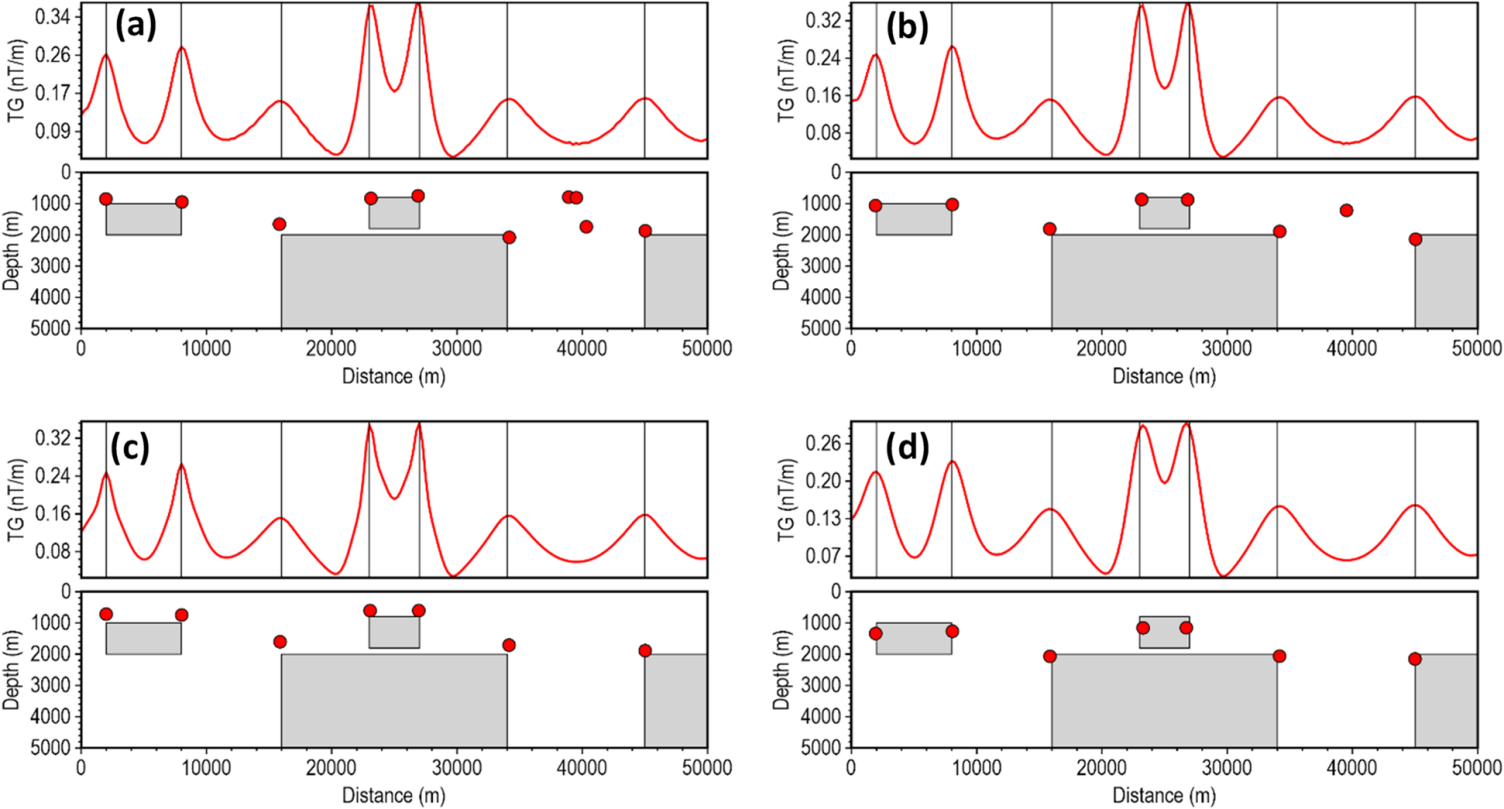
Source locations and depth estimates from the magnetic model response with **0**.1 nT Gaussian noise, computed using (**a**) FFT-TG, (**b**) ISVD-TG, (**c**) β-VDR-TG, and (**d**) β-VDR-with-β-HDR-TG.

**Fig. 7:**
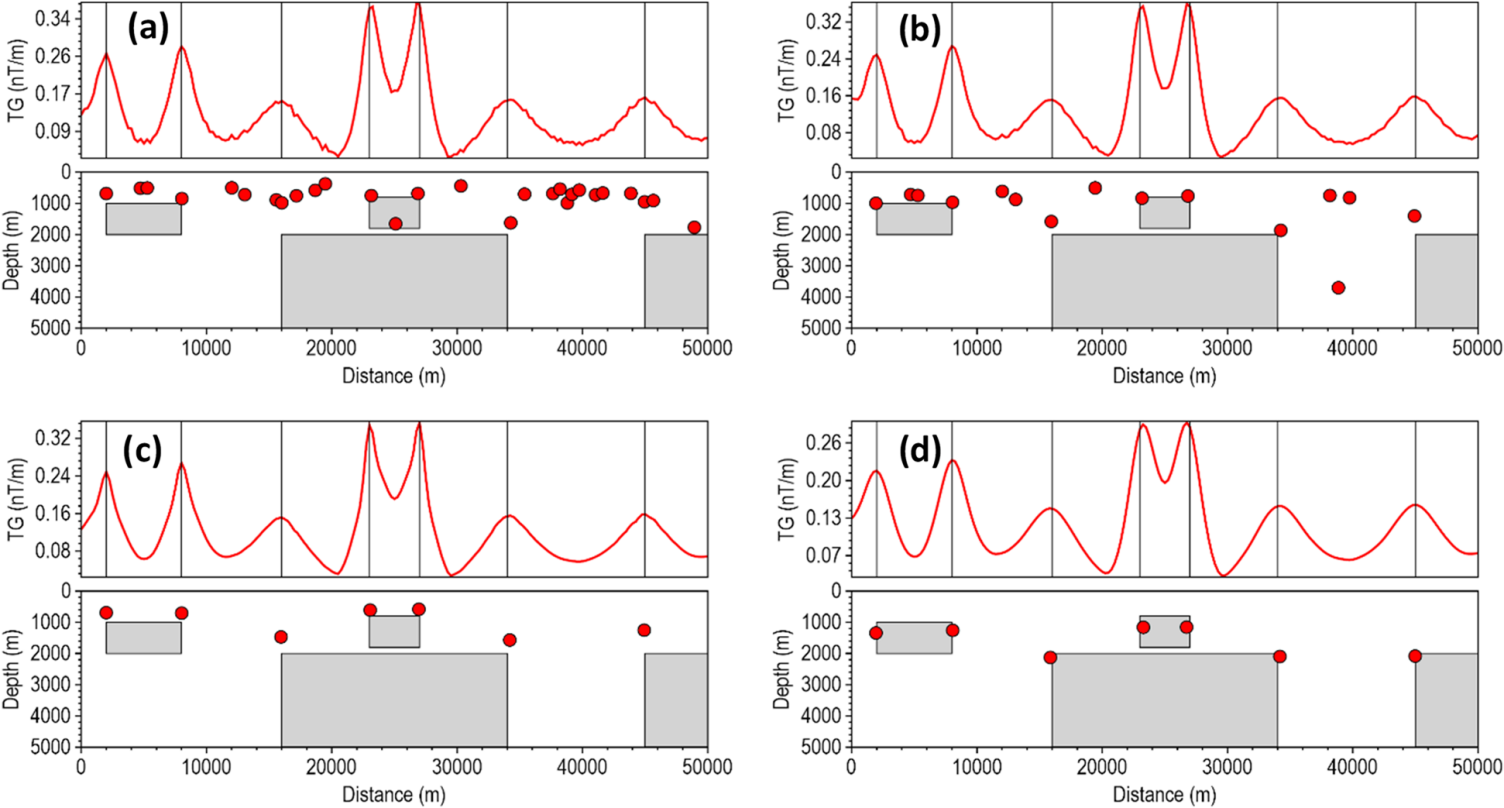
Source locations and depth estimates from the magnetic model response with 0.5 nT Gaussian noise, computed using (**a**) FFT-TG, (**b**) ISVD-TG, (**c**) β-VDR-TG, and (**d**) β-VDR-with-β-HDR-TG.

**Fig. 8:**
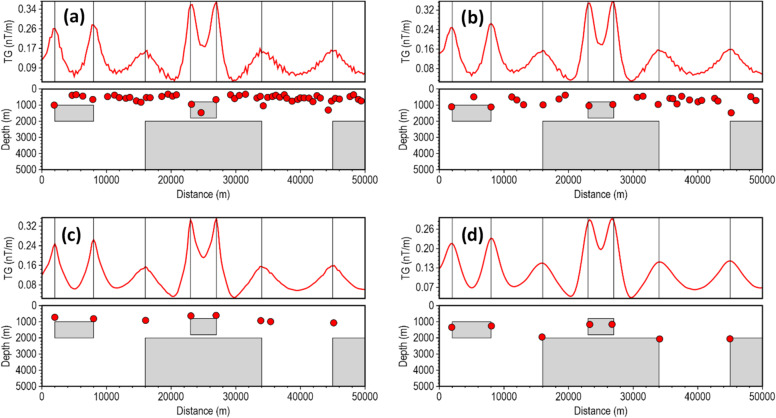
Source locations and depth estimates from the magnetic model response with 1.0 nT Gaussian noise, computed using (**a**) FFT-TG, (**b**) ISVD-TG, (**c**) β-VDR-TG, and (**d**) β-VDR-with-β-HDR-TG.

**Fig. 9:**
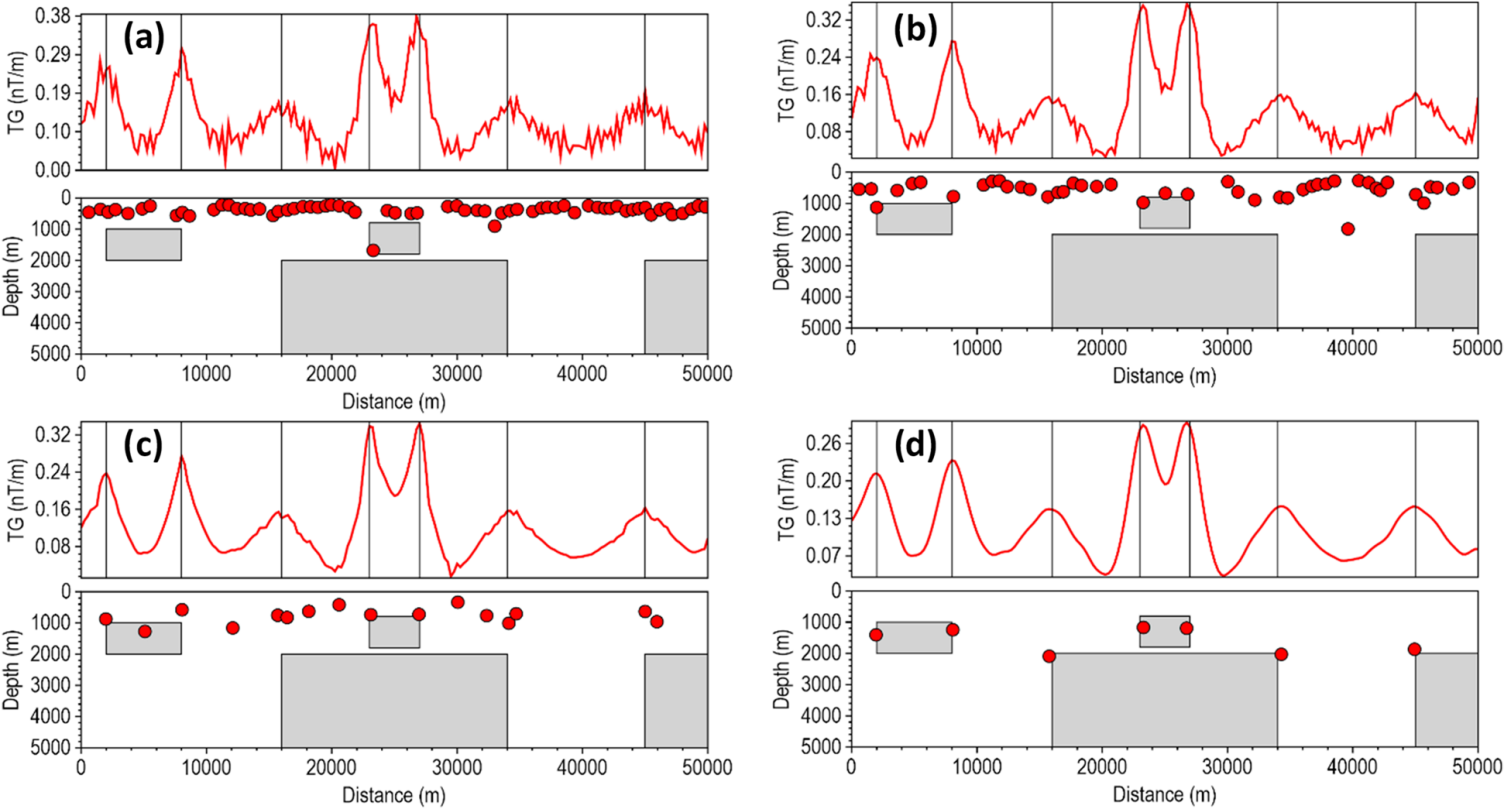
Source locations and depth estimates from the magnetic model response with 3.0 nT Gaussian noise, computed using (**a**) FFT-TG, (**b**) ISVD-TG, (**c**) β-VDR-TG, and (**d**) β-VDR-with-β-HDR-TG.

**Fig. 10:**
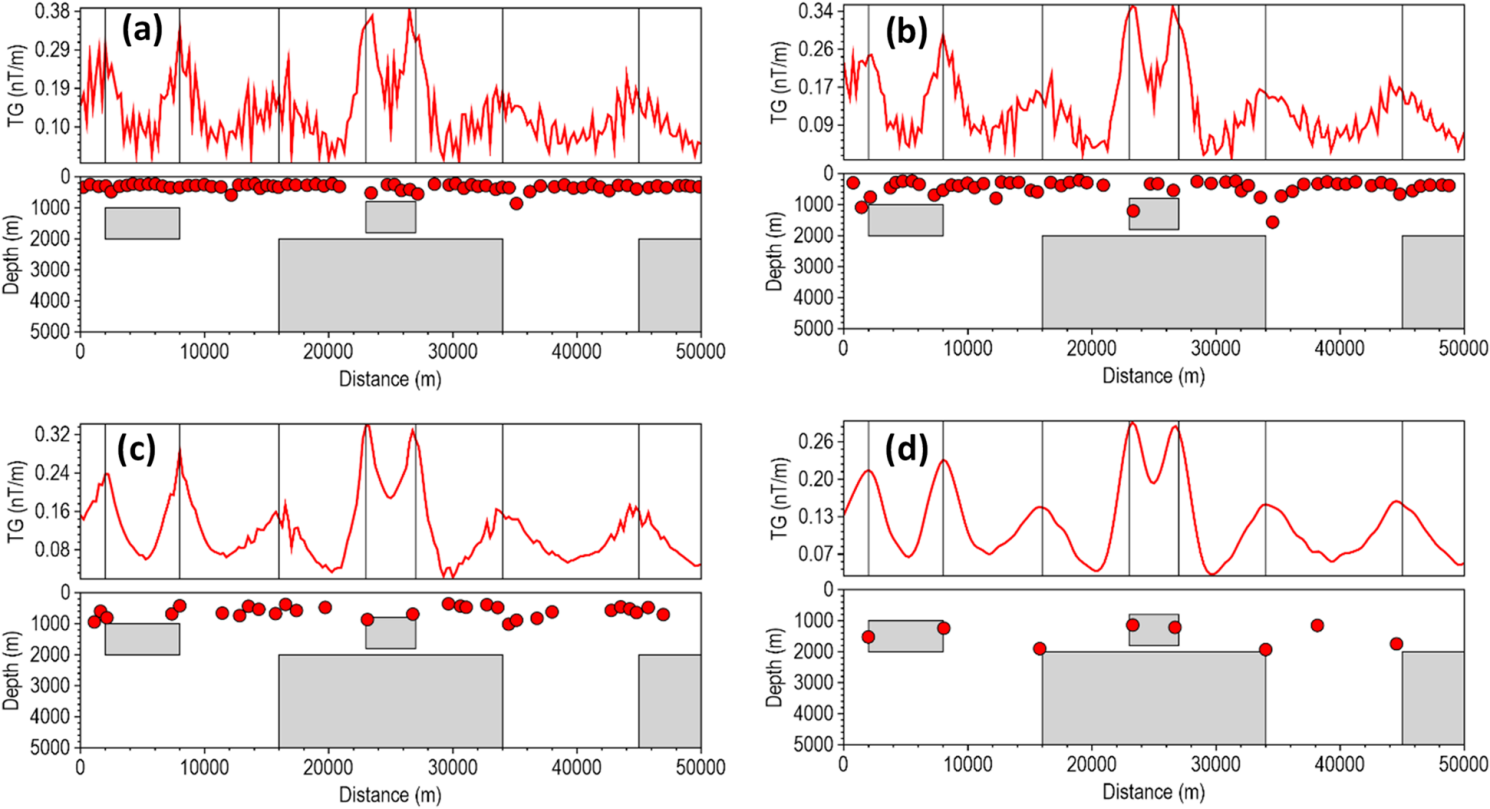
Source locations and depth estimates from the magnetic model response with 5.0 nT Gaussian noise, computed using (**a**) FFT-TG, (**b**) ISVD-TG, (**c**) β-VDR-TG, and (**d**) β-VDR-with-β-HDR-TG.

Most depth estimation methods rely heavily on the local curvature of derivative-dependent special functions (such as the TG) for accurate depth estimation^22^. Instability in the derivatives introduces high-frequency oscillations into the special function profile, artificially creating false peaks and inflating the local curvature near the true peak, and thus, results in inaccurate location and depth solutions for less robust methods in noise-contaminated data. We assessed the stability of the β-VDR-with-β-HDR method for source edge detection and depth estimation with Gaussian noise having standard deviations of 0.1 nT, 0.5 nT, 1.0 nT, 3.0 nT and 5.0 nT (Figs. 6, 7, 8, 9 and 10, respectively). The depths were calculated using a structural index of zero. The red line plot represents the TG profile, while the red circles represent the source solutions (locations and depths).

For the low noise level (0.1 nT), all methods peaked over the source edges and provided good depth estimates, as shown in Fig. 6. The FFT-TG (Fig. 6a) and ISVD-TG (Fig. 6b), however, included three and one false solutions, respectively, while the β-VDR-TG and the β-VDR-with-β-HDR-TG had no false solutions. The results of this test showed that the ISVD-TG is more stable than the FFT-TG for source edge detection and depth estimation.

As the noise increased to 0.5 nT (Fig. 7), the FFT-TG and ISVD-TG became more unstable with many spurious solutions, while the β-VDR-TG and the β-VDR-with-β-HDR-TG remained stable. Some β-VDR-TG depths were, however, underestimated at some edges, while β-VDR-with-β-HDR-TG still provided more reliable depth solutions. At 1.0 nT noise level (Fig. 8), the FFT-TG was highly unstable, and the ISVD-TG number of spurious solutions increased with many underestimated depths. The β-VDR-TG, showing only one false solution, was stable with accurate edge locations but underestimated depth solutions at some source edges. The β-VDR-with-β-HDR-TG remained stable with reliable edge and depth estimates.

At 3 and 5 nT noise levels (Figs. 9 and 10, respectively), the FFT-TG and ISVD-TG solutions became virtually unusable, having numerous false locations and depth solutions. The β-VDR-TG at these noise levels performed better than FFT-TG and ISVD-TG, but also produced many false solutions with highly underestimated depths at most edges. In contrast, the β-VDR-with-β-HDR-TG remained stable in the presence of these high-level noise conditions, with the location and depth solutions still reliable. The β-VDR-with-β-HDR-TG gave no false solution at 3 nT noise level and only one at 5 nT noise level.

**Fig. 11:**
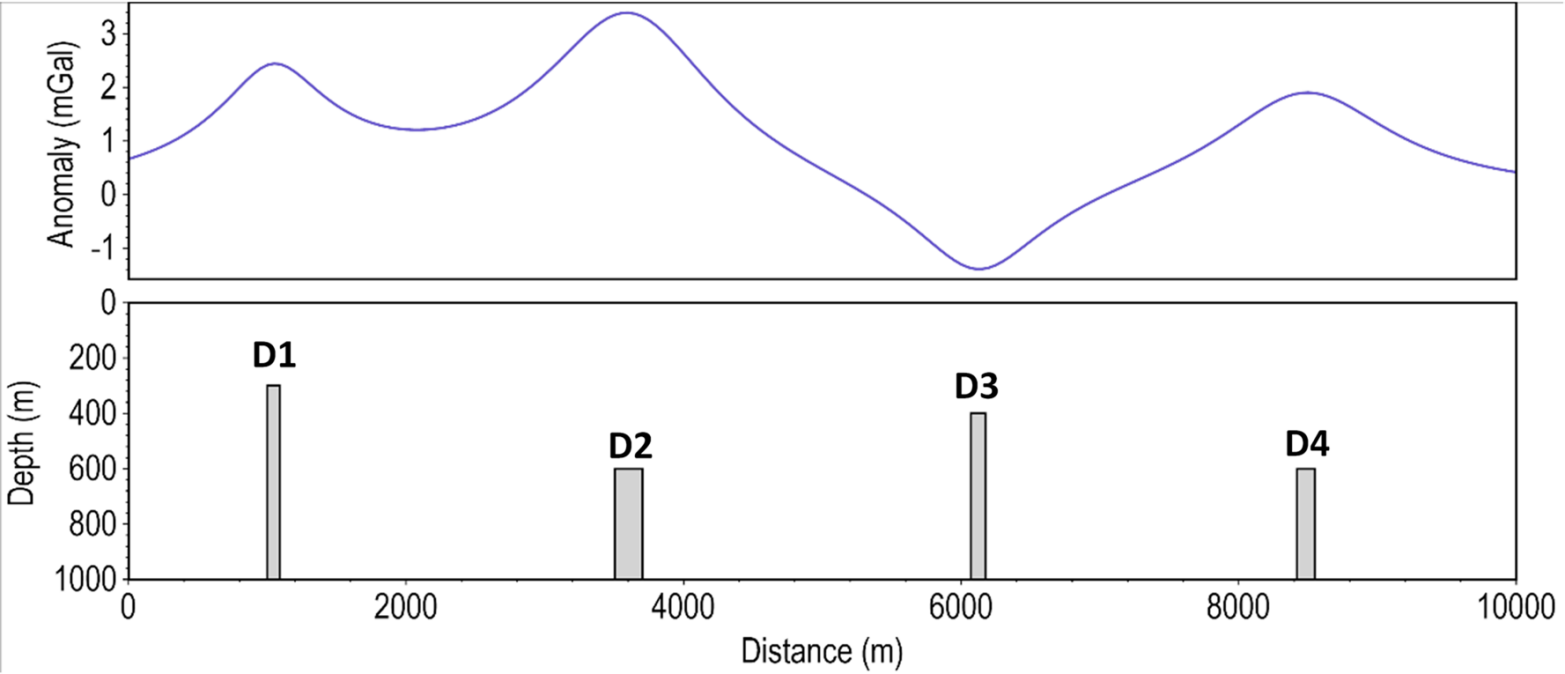
2D synthetic gravity model.

We also conducted synthetic gravity anomaly tests to evaluate the performance of the β-VDR-with-β-HDR on gravity profile data. For these tests, we considered a model consisting of four vertical dikes (labelled D1-D4) with different densities, dimensions, and positions. The model parameters are presented in Table 2, and the spatial arrangement of the dikes and their corresponding gravity responses are shown in Fig. 11.

**Fig. 12:**
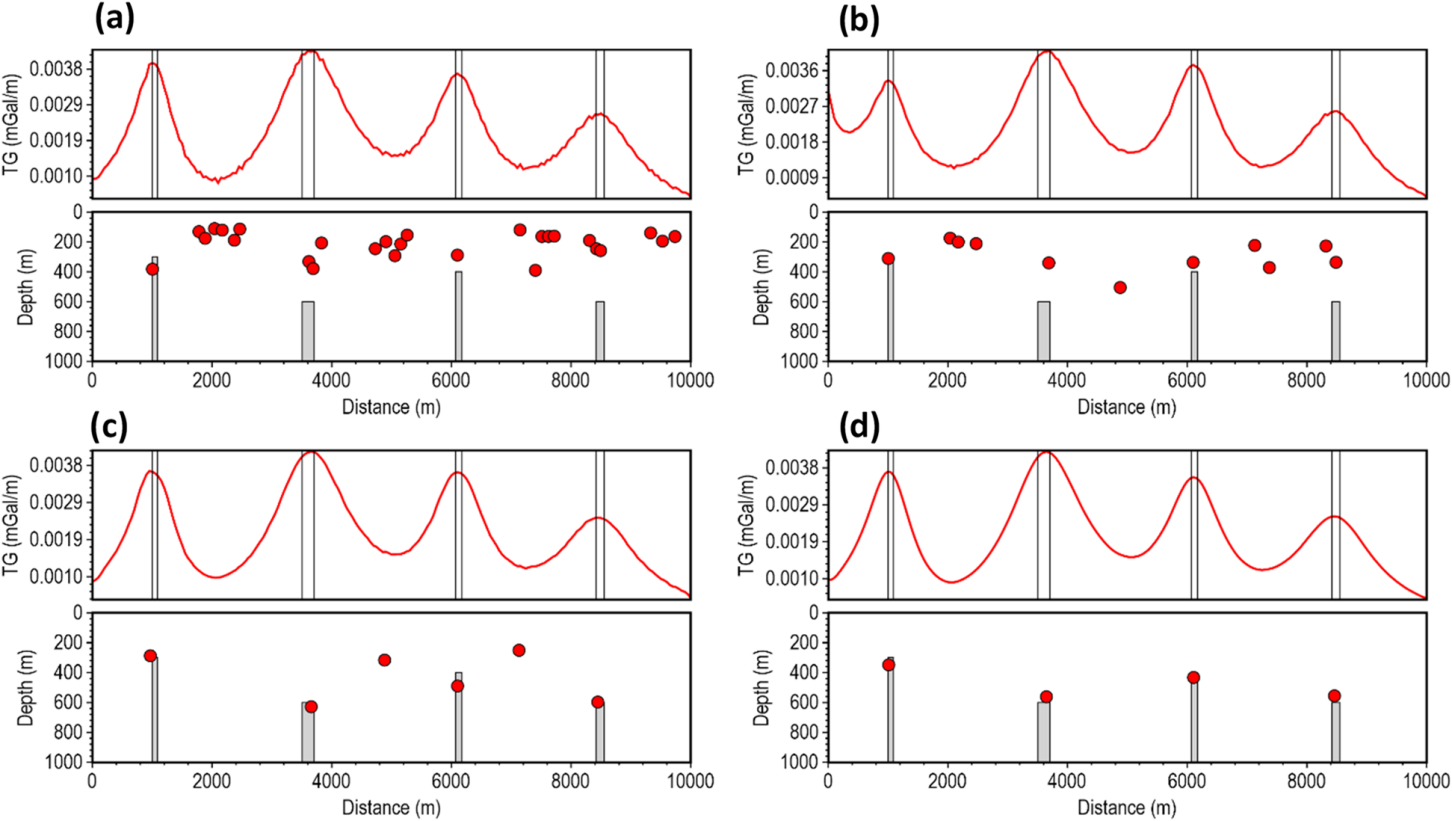
Source locations and depth estimates from the gravity model response corrupted with 0.001 mGal Gaussian noise, computed using (**a**) FFT-TG, (**b**) ISVD-TG, (**c**) β-VDR-TG, and (**d**) β-VDR-with-β-HDR-TG.

**Fig. 13:**
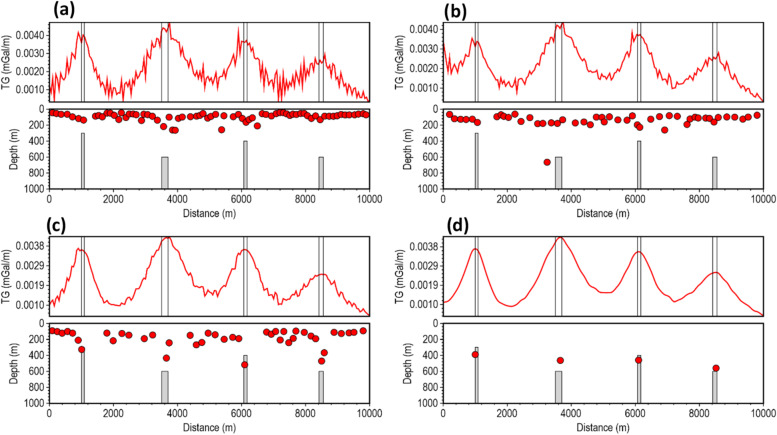
Source locations and depth estimates from the gravity model response corrupted with 0.01 mGal Gaussian noise, computed using (**a**) FFT-TG, (**b**) ISVD-TG, (**c**) β-VDR-TG, and (**d**) β-VDR-with-β-HDR-TG.

**Fig. 14:**
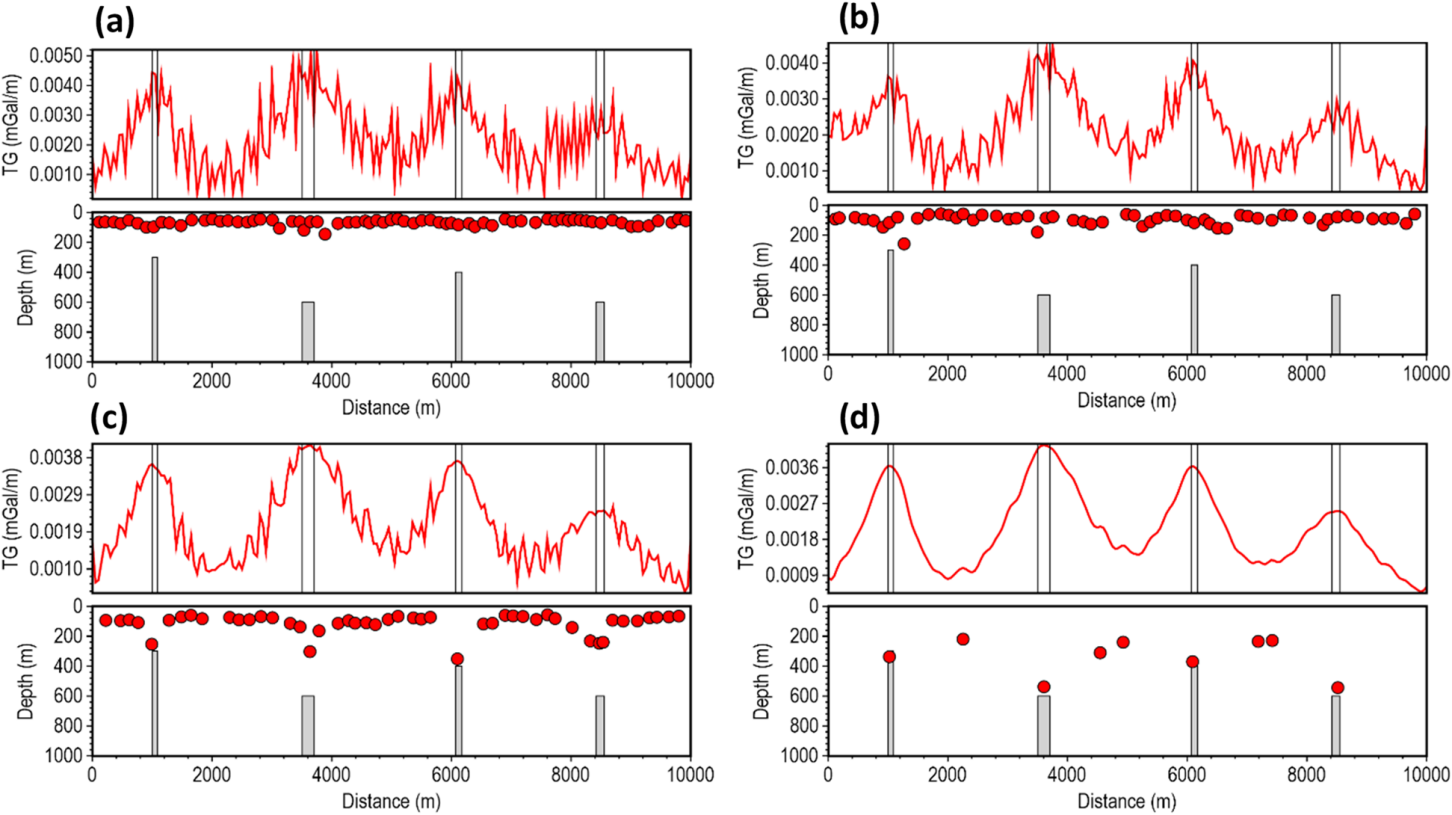
Source locations and depth estimates from the gravity model response corrupted with 0.02 mGal Gaussian noise, computed using (**a**) FFT-TG, (**b**) ISVD-TG, (**c**) β-VDR-TG, and (**d**) β-VDR-with-β-HDR-TG.

The performance of the β-VDR-with-β-HDR method was tested at different Gaussian noise levels with standard deviations of 0.001, 0.01, and 0.02 mGal (approximately 0.03%, 0.3% and 0.6% of the maximum anomaly amplitude, respectively). Similar to the magnetic test results, the gravity tests (Figs. 12, 13 and 14) showed a clear hierarchy in method performance: β-VDR-with-β-HDR performed better than β-VDR, β-VDR outperformed ISVD, and ISVD outperformed FFT. Even at a low noise level (0.001 mGal Gaussian noise), the ISVD-TG and FFT-TG location and depth solutions contained many spurious solutions, demonstrating the high noise sensitivity of the derivative operators and curvature analysis.

At higher noise levels (0.01 and 0.02 mGal), the performance gap widened significantly. Both ISVD and FFT became highly unstable, producing completely unusable solutions. The β-VDR also showed signs of instability, with numerous false solutions and underestimated depths. In contrast, the β-VDR-with-β-HDR method maintained stable, geologically consistent results with reliable depth estimates across the noise levels, and produced very few spurious solutions at the 0.02 mGal noise level, where others failed.

**Table 2:** Densities and geometric parameters of four 2D dikes.

Dike	Density contrast (g/cm^3^)	Top (m)	Bottom (m)	Left (m)	Right (m)
D1	1.5	300	1000	1000	1090
D2	2.5	600	1000	3505	3703
D3	− 1.5	400	1000	6070	6175
D4	2.2	600	1000	8420	8550

The results of the 2D tests clearly show the superior stability of β-VDR-with-β-HDR over the original β-VDR method for robust qualitative and quantitative interpretation of potential field profile. Although the β-VDR method performed significantly better than the ISVD and standard FFT methods, combining its vertical derivative with a less robust FD horizontal derivative limits its effective robustness for profile data interpretation. The β-VDR-with-β-HDR-TG, on the other hand, provides a balanced robustness across all derivatives, producing smooth, well-defined special function (such as TG) peaks, yielding reliable depth estimates, even in highly contaminated data.

### 3D synthetic model tests

**Fig. 15:**
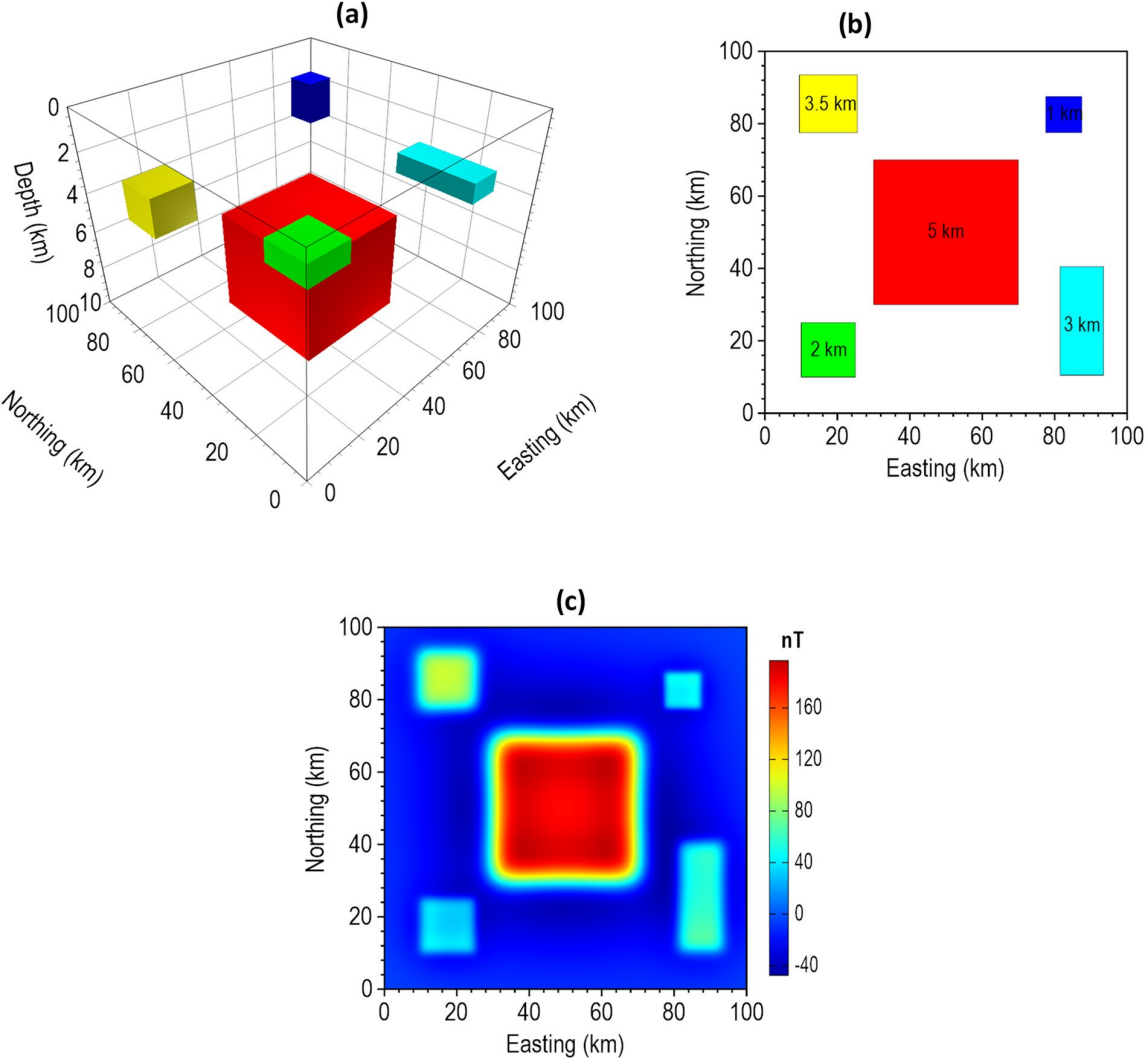
The 3D magnetic synthetic model. The (**a**) 3D view (**b**) plan view, and (**c**) magnetic anomaly of the model.

For the 3D synthetic tests, we started with a magnetic model. The model includes five prismatic magnetic sources having different positions, dimensions and magnetization property as listed in Table 3 and visually presented in Fig. 15. Induced magnetization with inclination of $\:90^\circ\:$ and declination of $\:0^\circ\:$ is assumed. The complete model parameters are presented in Table 3. The 3D view, plan view and magnetic anomaly of the model are displayed in Fig. 15a–c, respectively.

**Table 3:** 3D magnetic model parameters.

Model parameter	Body 1 (Red)	Body 2 (Green)	Body 3 (Cyan)	Body 4 (Blue)	Body 5 (Yellow)
x-coordinates of center (km)	50	17.5	87.5	82.5	17.5
y-coordinates of center (km)	50	17.5	25.5	82.5	85.5
Top depth (km)	5	2	3	1	3.5
Width (km)	40	15	12	10	16
Length (km)	40	15	30	10	16
Height (km)	5	1	1	2	2
Magnetization (A/m)	1.5	0.7	1.3	0.3	1.1

**Fig. 16:**
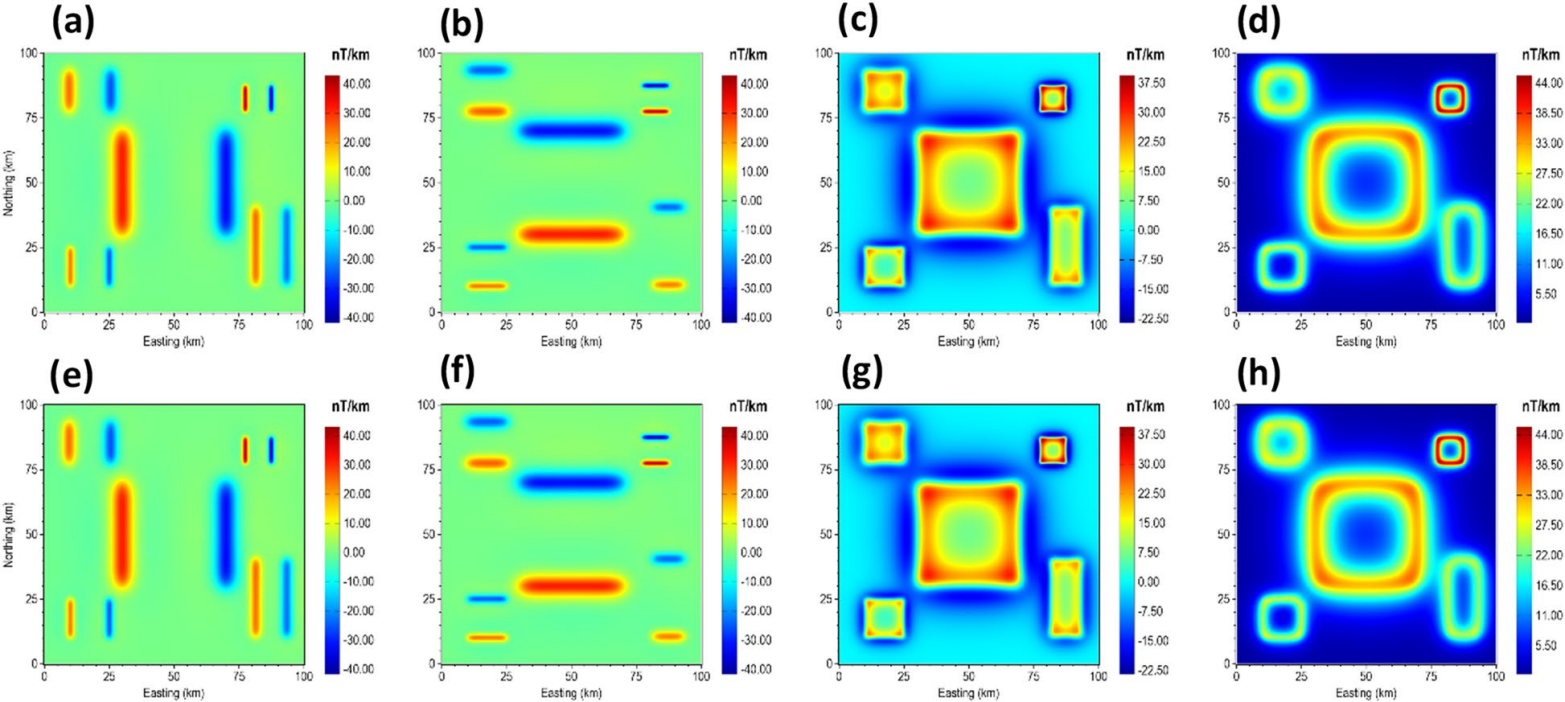
Derivatives of the magnetic anomaly of the 3D model. The panels show FFT (**a**–**d**) and β-VDR-with-β-HDR (**e**–**h**) results, specifically: x-derivative (**a**,**e**), y-derivative (**b**,**f**), vertical derivative (**c**,**g**), and TG (**d**,**h**).

To demonstrate the correctness of the proposed β-VDR-with-β-HDR method for gridded potential field data, we performed a direct comparison of the β-VDR-with-β-HDR with standard frequency domain filters. This was achieved by setting the stabilization parameter to zero (β = 0), which theoretically reduces the β-VDR-with-β-HDR operators to standard derivative operators. We computed the x-derivative, y-derivative, vertical derivative, and total gradient (TG) of the magnetic anomaly of the 3D model for the comparison. The results, presented in Fig. 16, verify the mathematical accuracy and consistency of the β-VDR-with-β-HDR method, whose derivatives are in close agreement with those obtained from the FFT, with RMSE values of 3.43 × 10⁻^5^, 3.43 × 10⁻^5^, 4.84 × 10⁻^5^, and 4.87 × 10⁻^5^ nT/m for the x-derivative, y-derivative, vertical derivative, and TG, respectively. The near-zero RMSE values confirm that the β-VDR-with-β-HDR reproduces the standard FFT results without stabilization, which is a prerequisite for ensuring that the compact β-VDR and the β-HDR preserves the fundamental mathematical properties of derivative operators.

**Fig. 17:**
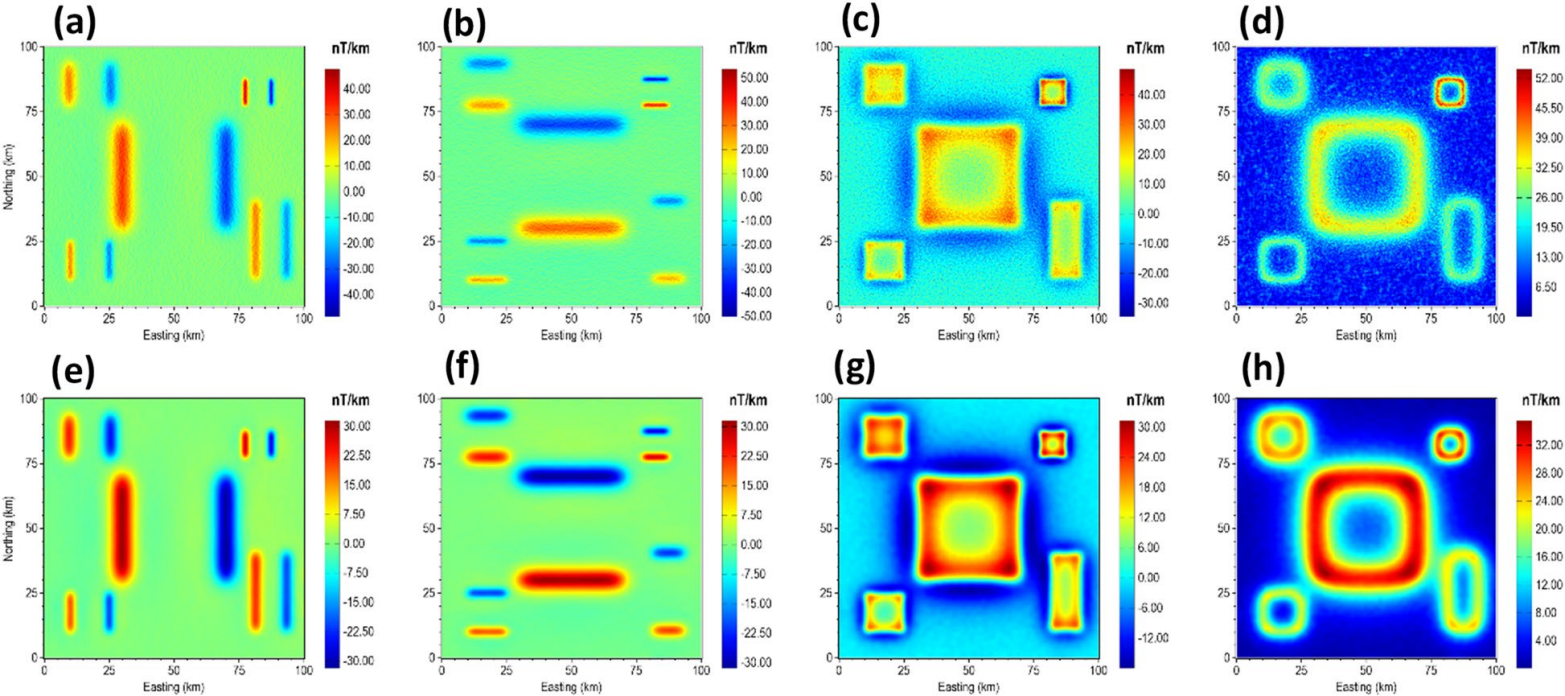
Derivatives of the magnetic anomaly of the 3D model corrupted with 1 nT Gaussian noise. The panels show FFT (**a**–**d**) and β-VDR-with-β-HDR (**e**–**h**) results, specifically: x-derivative (**a**,**e**), y-derivative (**b**,**f**), vertical derivative (**c**,**g**), and TG (**d**,**h**).

In the presence of noise, the advantages of the β-VDR-with-β-HDR become more evident. Under Gaussian noise with standard deviation of 1.0 nT, Fig. 17 illustrates that β-VDR-with-β-HDR derivatives exhibit robustness compared to FFT filters for the same set of derivatives. As noticeable in the TG maps, the β-VDR-with-β-HDR maintains smooth and reliable responses, while the FFT reflects lower signal-to-noise-ratio.

We compared the execution-time performance of the compact β-VDR (our proposed method) against the original β-VDR computation on a personal computer to determine the speed-up achieved by the compact formulation. Although the execution time depends on the computing resources, data size, data-preparation steps, and FFT algorithm used, the speed-up factor should remain comparable across systems. For our tests, we used a data grid (Fig. 15c) consisting 201 × 201 data points, and a Fourier transformation procedure involving padding the grid by at least 10% to the next power of two and filling the padded regions using the Maximum Entropy prediction^20,21^, such that the data is smoothly periodic across opposite edges to minimize spectral leakage, and then transforming the prepared data into the frequency domain using the Cooley–Tukey FFT algorithm^23^. For the original β-VDR, the required five upward continuations were computed first using separate Fourier transforms, and then using a faster implementation requiring only one forward FFT and five inverse FFTs. For reliable comparison, each test was repeated five times, and the average was determined. As summarized in Table 4, the compact β-VDR outperformed the original β-VDR by an average factor of 5.32 when compared with the initial implementation, and by a factor of 2.47 when compared with the faster implementation. These results demonstrate that the compact β-VDR substantially reduces computational cost, an advantage that becomes increasingly important when processing large datasets.

**Table 4:** Comparison of execution time for the proposed compact β-VDR and the original β-VDR.

Test	Original (ms)	Faster original (ms)	Proposed (ms)	Speed-up (proposed vs. original)	Speed-up (proposed vs. faster original)
1	1590	682	272	5.85	2.51
2	1417	871	270	5.25	3.23
3	1544	658	340	4.54	1.94
4	1532	631	269	5.7	2.35
5	1513	682	277	5.46	2.46
Average	1519.2	704.8	285.6	5.32	2.47

**Fig. 18:**
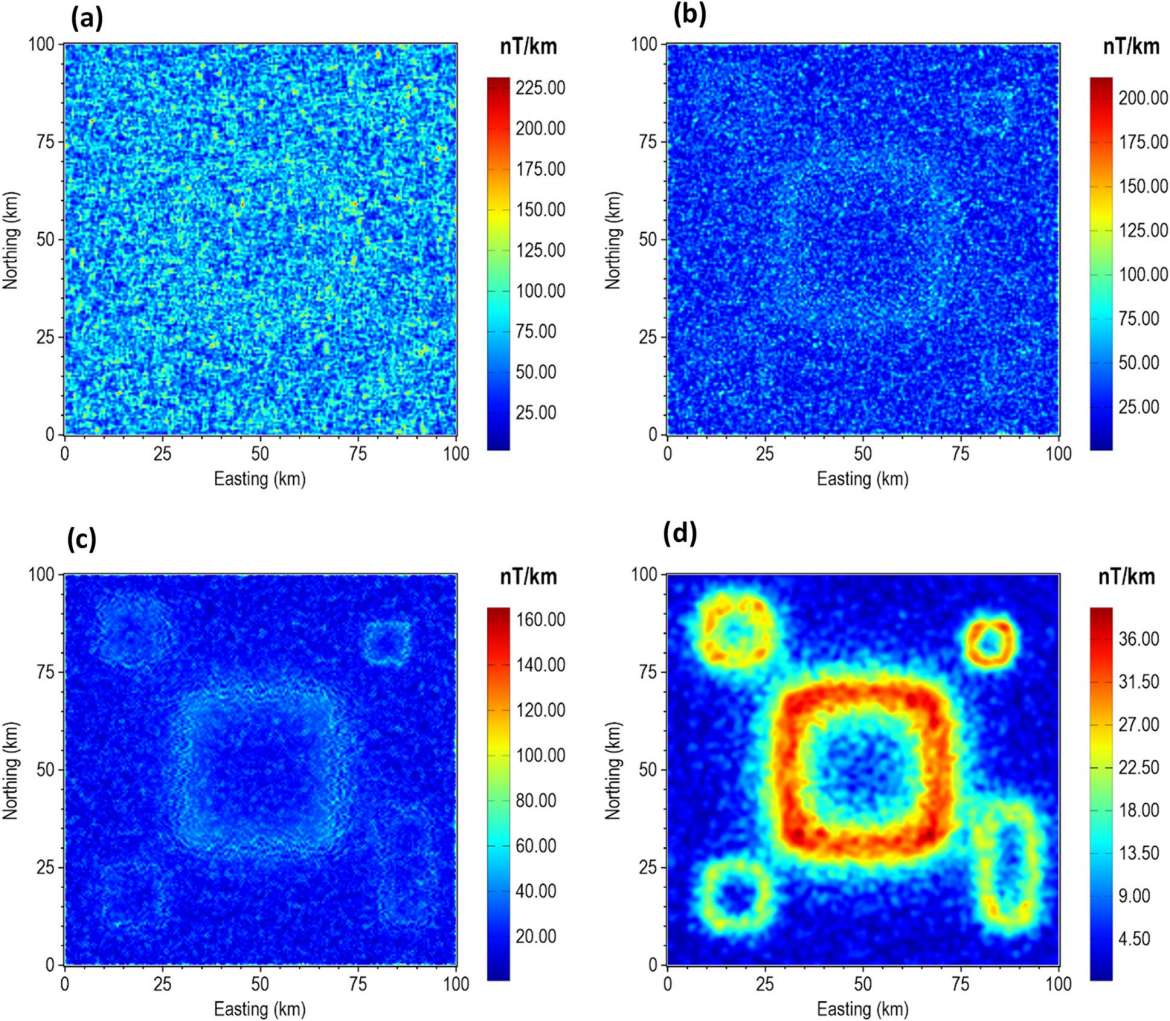
(**a**) FFT-TG (**b**) ISVD-TG (**c**) β-VDR-TG and (**d**) β-VDR-with-β-HDR-TG of the magnetic anomaly of the 3D model corrupted with 10 nT Gaussian noise.

Furthermore, we tested the robustness of β-VDR-with-β-HDR with FFT, ISVD and β-VDR methods for source edge enhancement under an extreme noise condition by contaminating the magnetic anomaly of the 3D model with a Gaussian noise having a standard deviation of 10 nT (5% of the maximum anomaly amplitude). The results are presented in Fig. 18. As shown in Fig. 18a, the conventional FFT filter completely failed. The source edges in the FFT-TG image were completely masked by noise amplification, resulting in a map that is entirely uninterpretable. The ISVD and β-VDR performed better than the conventional FFT, but the source edges in the ISVG-TG and β-VDR images are faintly discernible. The β-VDR-with-β-HDR-TG, on the other hand, exhibits significantly discernible source edges. Furthermore, the map displays a lower overall amplitude range compared to the alternatives, indicating a robust suppression of high amplitude noise. This affirms the superior level of robustness of the β-VDR-with-β-HDR over the original β-VDR method.

**Fig. 19:**
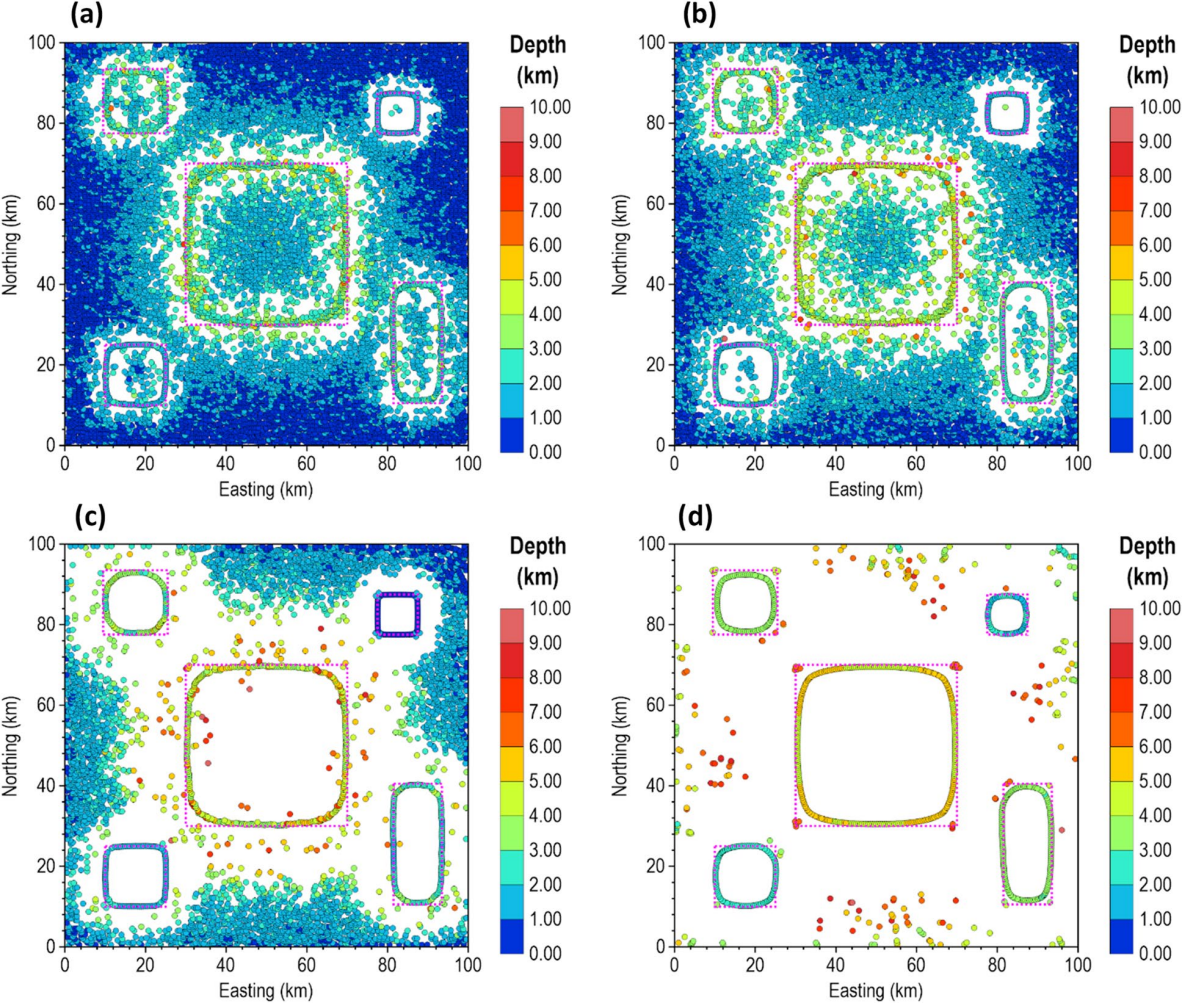
Source locations and depth estimates from (**a**) FFT-TG (**b**) ISVD-TG (**c**) β-VDR-TG and (**d**) β-VDR-with-β-HDR-TG of the magnetic anomaly of the 3D model corrupted with 0.1 nT Gaussian noise. The true edges of the sources are indicated by dotted rectangles.

**Fig. 20:**
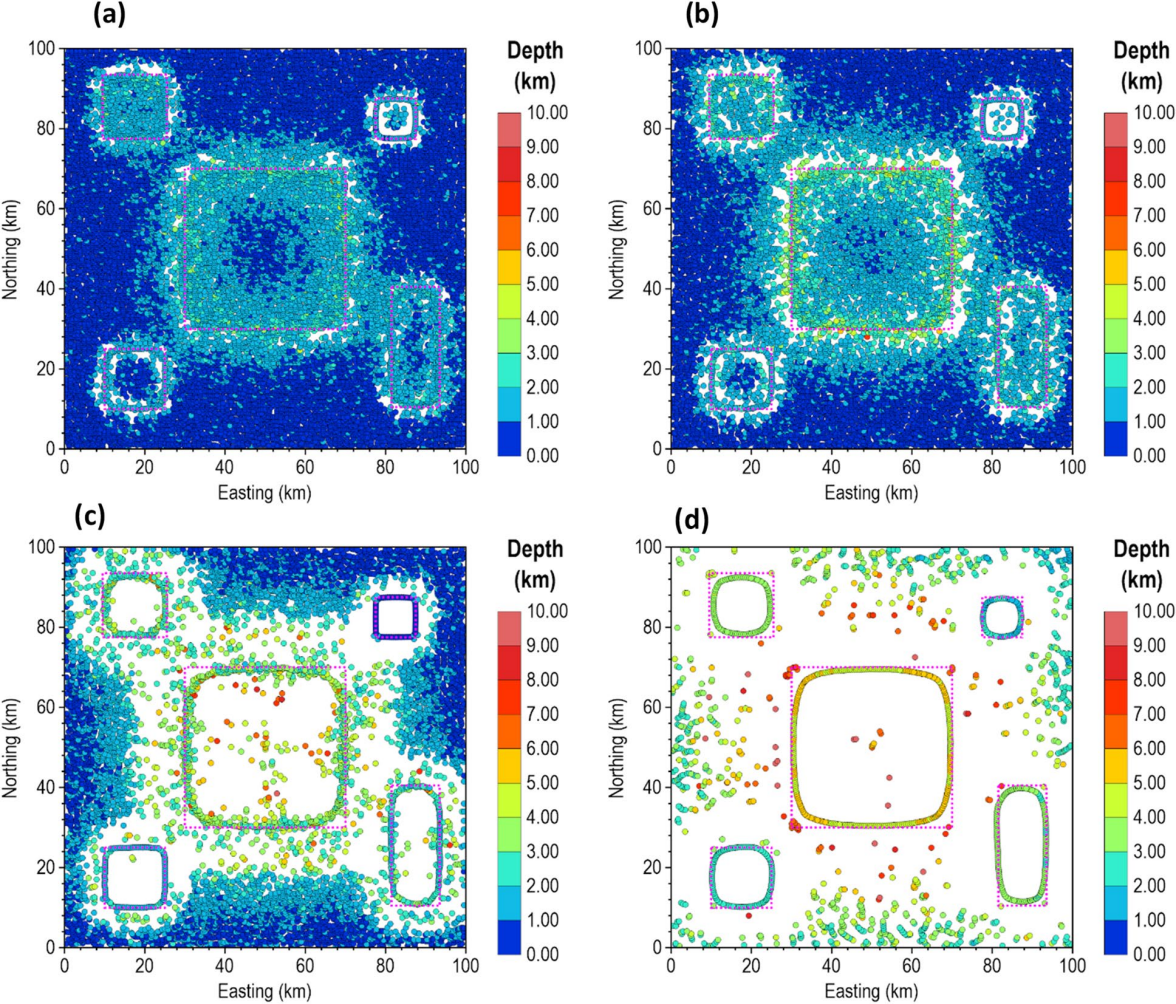
Source locations and depth estimates from (**a**) FFT-TG (**b**) ISVD-TG (**c**) β-VDR-TG and (**d**) β-VDR-with-β-HDR-TG of the magnetic anomaly of the 3D model corrupted with 0.3 nT Gaussian noise. The true edges of the sources are indicated by dotted rectangles.

We estimated the locations and depths of the 3D sources under Gaussian noise levels with standard deviations of 0.1 and 0.3 nT to test the reliability of the different methods. The standard deviations of these noise levels correspond to 0.051% and 0.153% of the maximum anomaly amplitude, respectively. The results for the 0.1 nT noise are presented in Fig. 19, while those for 0.3 nT noise are shown in Fig. 20. As expected, the β-VDR-TG and β-VDR-with-β-HDR-TG showed lower sensitivity to noise than the FFT-TG and ISVD-TG. All of the methods produced solutions at the locations of the source edges, but the FFT-TG and ISVD-TG results contained numerous spurious solutions, reducing the reliability. Although the β-VDR-TG result contained many false solutions, their numbers were significantly fewer than those of the FFT-TG and ISVD-TG. The β-VDR-with-β-HDR-TG produced clearer, well-defined source edge solutions with very few false solutions at both noise levels. The reliable solutions formed ridges along the source edges, while the spurious solutions were scattered or isolated.

In terms of depth estimation, the ISVD-TG provided slightly more accurate depth estimates than the FFT-TG at the true source locations, but both methods consistently underestimated the true depths of the deepest source and most other sources. The β-VDR-TG and the β-VDR-with-β-HDR-TG, on the other hand, gave more reliable depth estimates, with the β-VDR-with-β-HDR-TG producing the most accurate depths along the edges of both the shallow and deep sources. The underestimation of the true depth by the β-VDR-TG, especially at the higher noise level of 0.3 nT, can be attributed to its unstable horizontal derivative component. This shows the importance of using a stabilized derivative filter for all components to effectively increase the signal-to-noise ratio in a consistent manner.

**Fig. 21:**
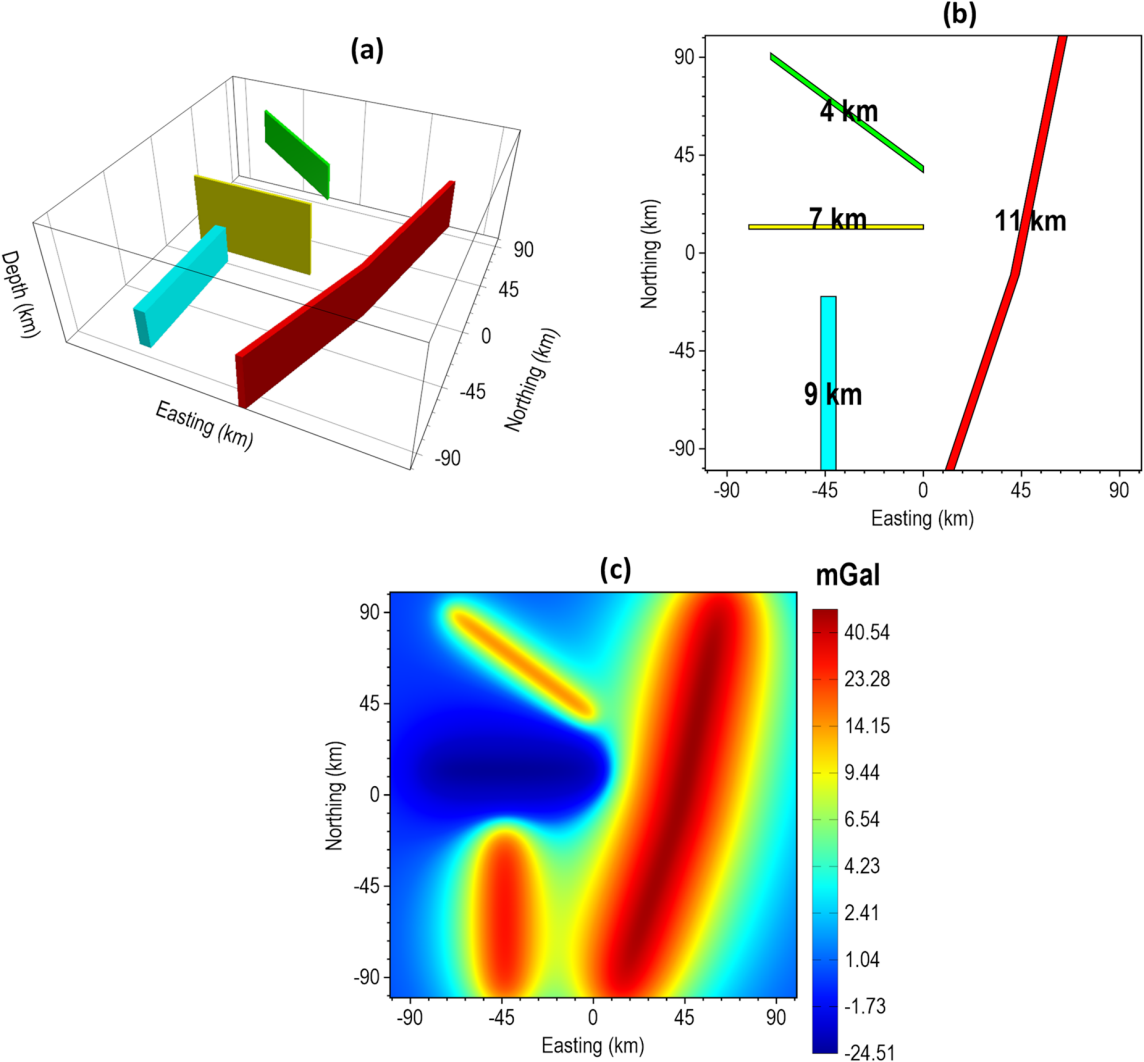
The 3D Synthetic Gravity Model. The (**a**) 3D view, (**b**) plan view, and (**c**) gravity anomaly of the model.

Furthermore, we evaluated the performance of our proposed method using a synthetic gravity anomaly model. Figure 21 shows the 3D view, top view, and the theoretical gravity response of the model. The model consists of dike-like bodies with different dimensions, orientations, density contrasts, and depths. The depth to the top of each body is indicated in Fig. 21b, and the parameters of the model are summarized in Table 5.

**Fig. 22:**
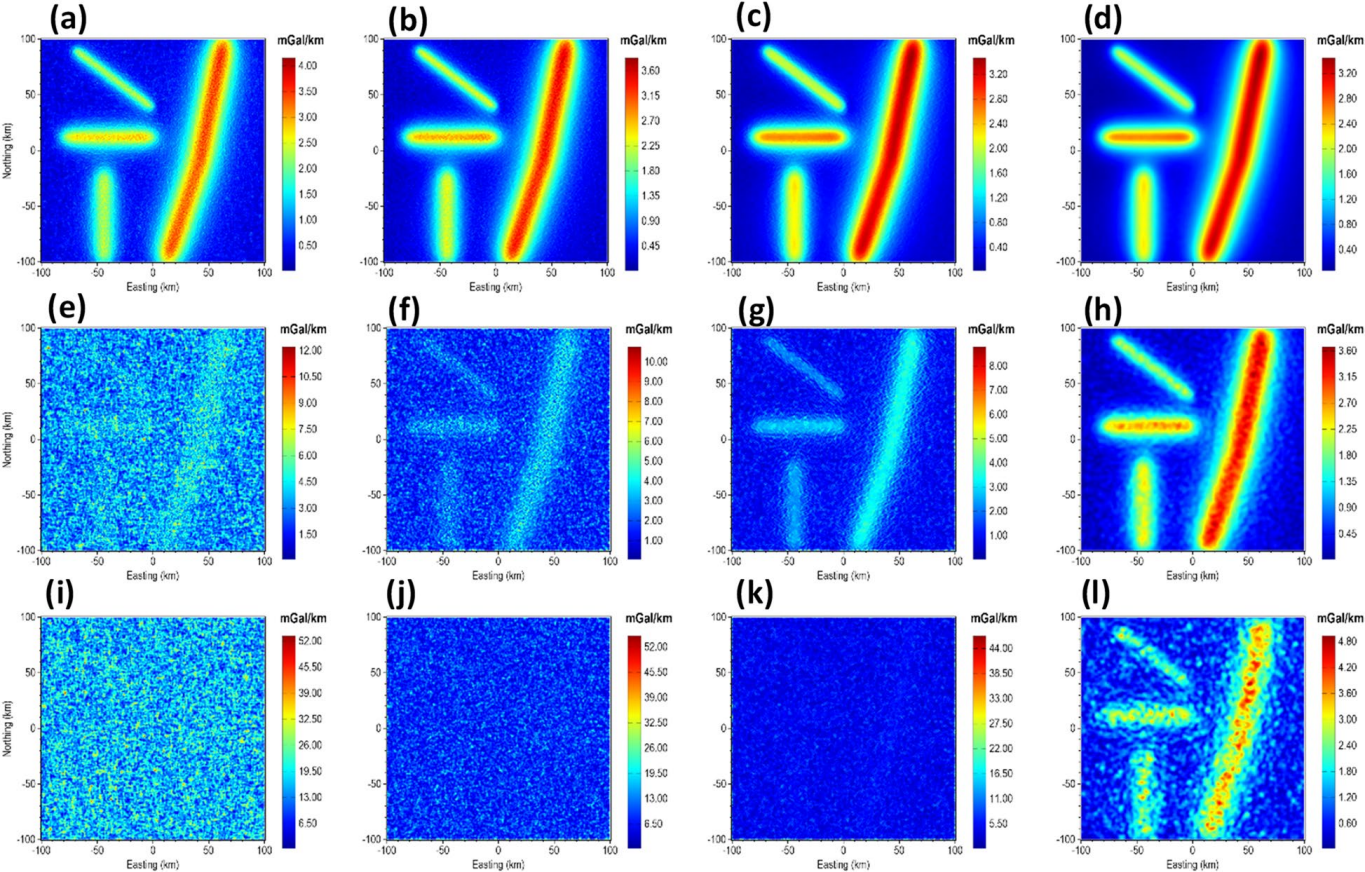
TG maps of the 3D gravity model corrupted with 0.1 mgal (**a**–**d**), 1.0 mgal (**e**–**h**) and 5.0 mGal (**i**–**l**). The panels show the results obtained from FFT-TG (**a**,**e**,**i**), ISVD-TG (**b**,**f**,**j**), β-VDR-TG (**c**,**g**,**k**) and β-VDR-with-β-HDR-TG (**d**,**h**,**l**) at the different noise levels.

Figure 22 illustrates the edge enhancement capabilities of β-VDR-with-β-HDR-TG compared with FFT-TG, ISVD-TG, and β-VDR-TG at different noise levels. When the model was corrupted with Gaussian noise having a standard deviation of 0.1 mGal (approximately 0.2% of the maximum anomaly amplitude), all of the methods produced clear edges of the anomalous sources, as shown in Fig. 22a–d. Although some noise artifacts were noticed in the results of FFT-TG, ISVD-TG and β-VDR-TG, the β-VDR-with-β-HDR-TG produced no discernible artifacts.

**Table 5:** The 3D gravity model parameters.

Model parameter	Body 1 (red)	Body 2 (cyan)	Body 3 (yellow)	Body 4 (green)
Top depth (km)	11	9	7	4
Height (km)	9	6	13	6
Width (km)	4	7	2	2.4
Length (km)	207	80	80	87
Density contrast (g/cm^3^)	1.5	0.6	-1.0	0.5

When the noise level was increased to 1.0 mGal (about 2% of the maximum anomaly amplitude), as shown in Fig. 22e, f, the β-VDR-TG became less stable but still outperformed ISVD-TG, which in turn performed better than FFT-TG. At this noise level, β-VDR-with-β-HDR-TG showed clear source edges, indicating its higher stability than the other methods.

Figure 22i–l show the response of the four methods to Gaussian noise with a standard deviation of 5.0 mGal (around 10% of the maximum anomaly amplitude). At this noise level, the FFT-TG, ISVD-TG and β-VDR-TG all failed with no traceable source edges. On the other hand, the β-VDR-with-β-HDR-TG still provided useful structural information, retaining identifiable source positions and orientations. These edge enhancement tests confirm that the β-VDR-with-β-HDR is more stable and reliable for edge detection in noisy gravity data. This stability demonstrates that using β-HDR along with β-VDR for source edge detection significantly enhances noise suppression while effectively preserving geological boundaries.

**Fig. 23:**
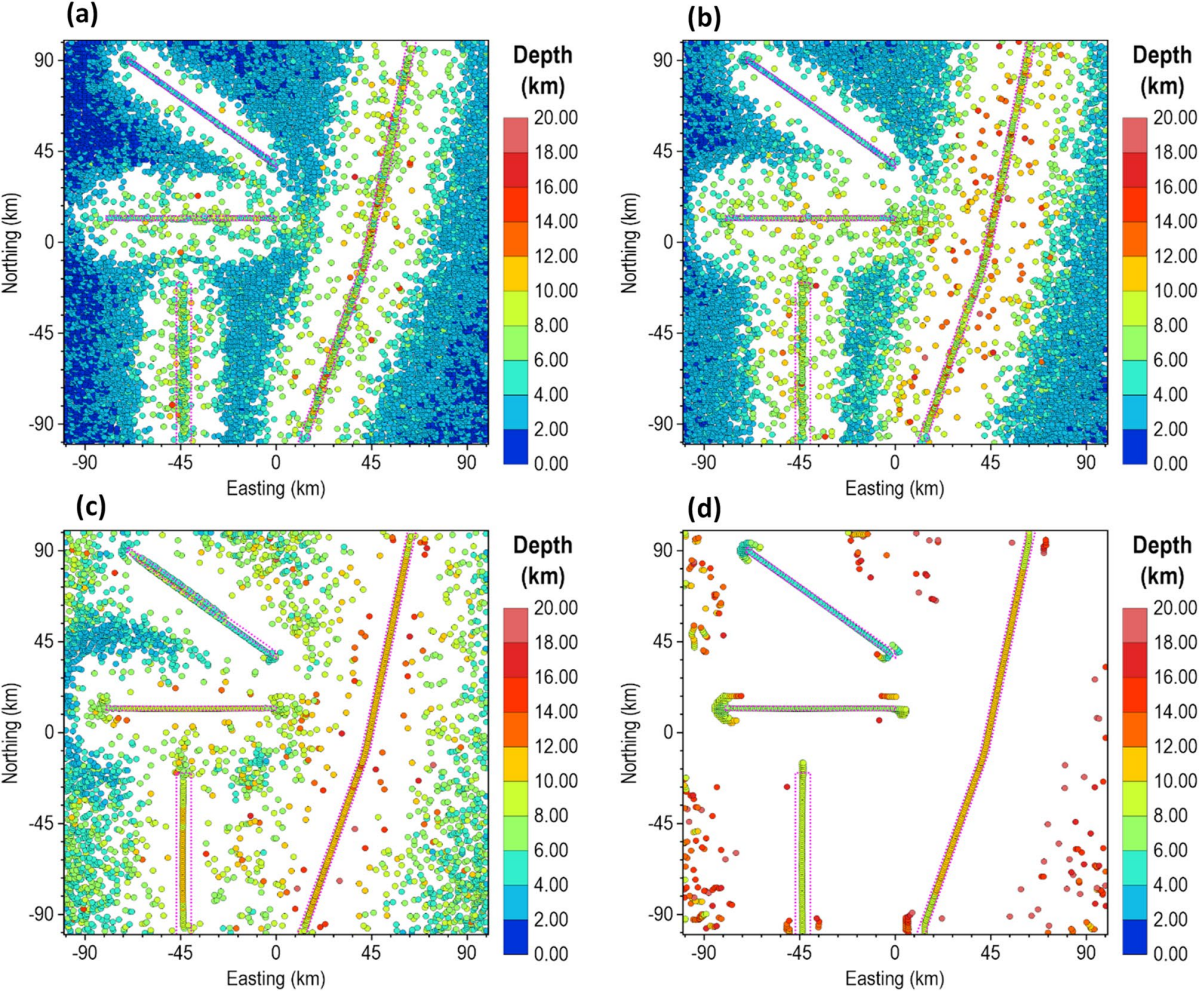
Source locations and depth solutions from (**a**) FFT-TG (**b**) ISVD-TG (**c**) β-VDR-TG and (**d**) β-VDR-with-β-HDR-TG of the 3D gravity anomaly model corrupted with Gaussian noise having a standard deviation of 0.01 mGal. The true locations of the sources are indicated by dotted polygons.

**Fig. 24:**
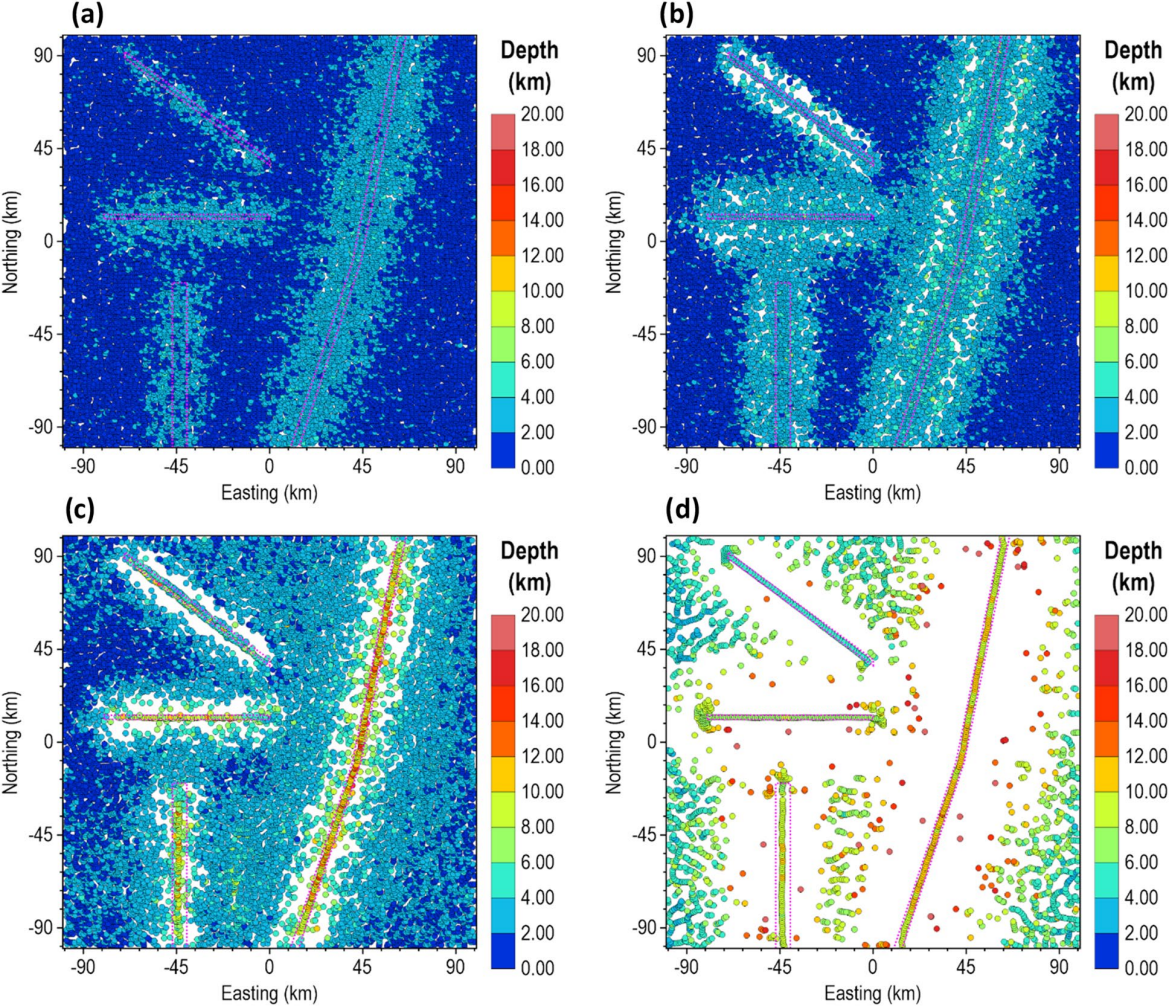
Source locations and depth solutions from (**a**) FFT-TG (**b**) ISVD-TG (**c**) β-VDR-TG and (**d**) β-VDR-with-β-HDR-TG of the 3D gravity anomaly model corrupted with Gaussian noise having a standard deviation of 0.1 mGal. The true locations of the sources are indicated by dotted polygons.

We also assessed the effectiveness of β-VDR-with-β-HDR-TG, β-VDR-TG, ISVD-TG and FFT-TG for source location and depth estimation under noisy gravity conditions. The results obtained are presented in Figs. 23 and 24 for Gaussian noise with standard deviations of 0.01 mGal (approximately 0.02% of the maximum anomaly amplitude) and 0.1 mGal (approximately 0.2% of the maximum anomaly amplitude), respectively. As expected, the β-VDR-with-β-HDR-TG gave more reliable location and depth estimates with fewer spurious solutions across all noise levels.

At the 0.01 mGal noise level (Fig. 23), all methods captured the locations and main trends of the sources, but FFT-TG and ISVD-TG produced numerous spurious solutions and underestimated some source depths. The β-VDR-TG performed better but still produced significant spurious solutions. The depth estimates by β-VDR-with-β-HDR-TG agreed with the true values and were more reliable than those of β-VDR-TG. When the noise was increased to 0.1 mGal (Fig. 24), the performance differences became sharper. The β-VDR-with-β-HDR-TG solutions remained close to the true depth values and contained significantly fewer spurious solutions than the other methods.

The results from both the gravity and magnetic synthetic tests show that although the β-VDR improves robustness compared to traditional FFT- and ISVD-based approaches, its performance is still limited by the instability of the FD horizontal derivative component. By integrating the β-HDR, the generalized β-VDR-with-β-HDR method effectively overcomes this limitation, offering a more stable, accurate, and reliable solution for source edge detection and depth estimation under noisy conditions.

### Real world application

**Fig. 25:**
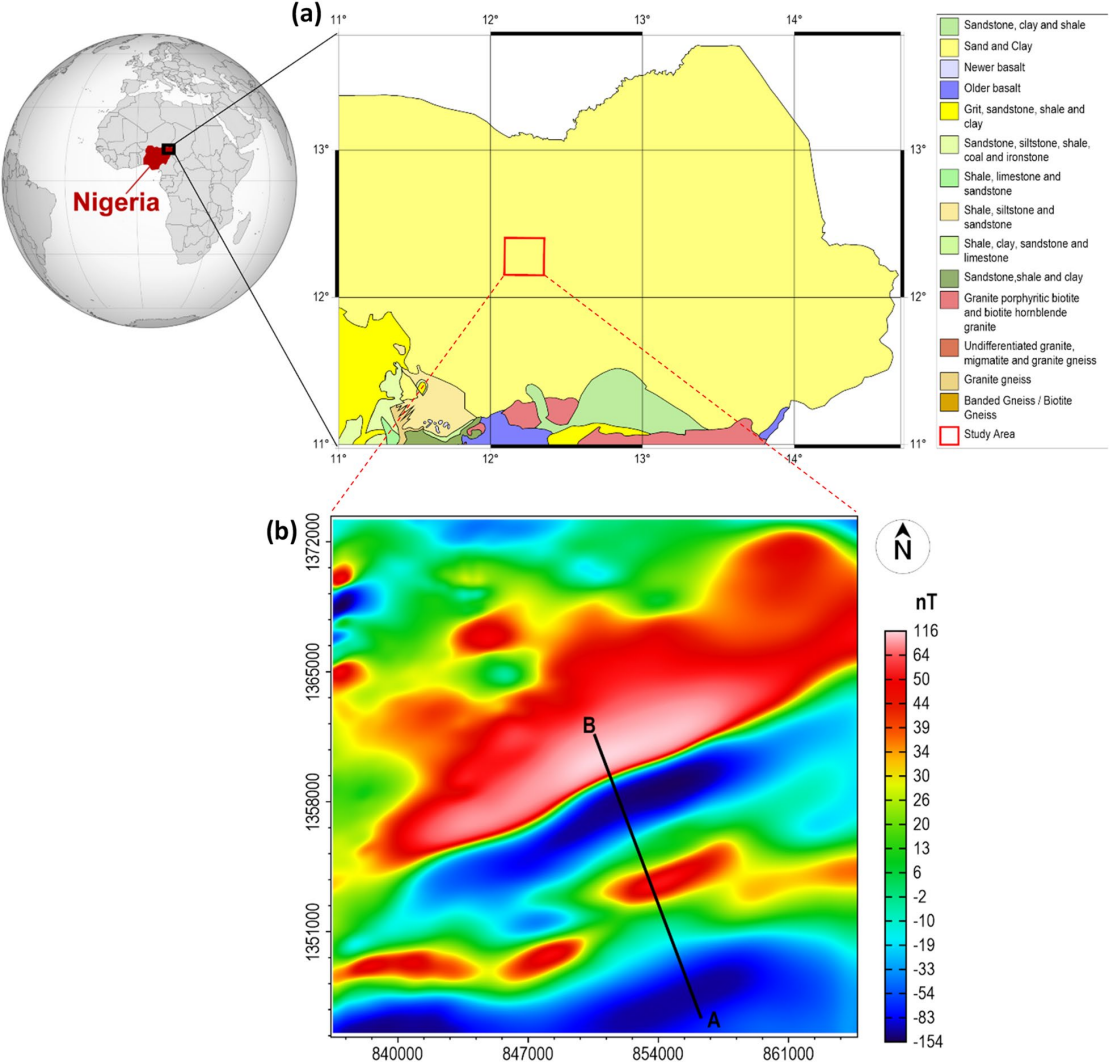
(**a**) The geological map of parts of North-East Nigeria showing the Chad Basin, Nigeria with the investigated area marked by a red rectangle. (**b**) The reduced-to-equator (RTE) magnetic anomaly map of the investigated area.

We applied the β-VDR-with-β-HDR derivatives for magnetic structural investigation of the Chungul Bulturi area within the Nigerian sector of the Chad Basin. The geological map of the study area and its environs is presented in Fig. 25a.

The Chad Basin is an intracontinental sedimentary-filled rift basin whose creation is genetically linked to the West and Central African Rift System (WCARS). This rifting episode occurred during the break-up of the Gondwana supercontinent and the opening of the South Atlantic Ocean in the Early Cretaceous (∼145–100 Ma)^24–26^. Stratigraphically, the topmost layer of the basin consists of Quaternary interbeds of sand and clay, which are collectively referred to as the Chad Formation. Beneath this formation are tertiary to cretaceous strata mainly comprising beds of sandstone, clay, siltstone, limestone, and shale. Tectonically, the basin’s evolution is categorized into four main stages^25–27^: the Pan African Crustal Consolidation (750–550 Ma), during which the major (NE–SW and possibly NNE-SSW trending) basement structures were formed; the Early Rift stage (130–98 Ma), which is the main rifting event that created the basin; the Late Rift stage (98–75 Ma), whose tectonic activities produced geological structures that include NW-SE trending faults and folds; and lastly, the Post Rift stage (66–0 Ma), which exhibited no significant tectonic events. The basin’s most significant tectonic events concluded during the Last Rift stage, and consequently, the overlying Tertiary and Quaternary strata have no notable faulting or significant folding. The majority of the faults in the basin resulted from basement fault propagations^28,29^. The presence of intrasedimentary intrusives and volcanic centers in the basins, which are relevant magnetic sources, has also been reported in the literature^30–33^.

The Chungul Bulturi area is one of the regions within the Nigerian sector of the Chad Basin suspected to contain geological structures hosting valuable geothermal resources or hydrocarbon fluids^31,34^. Previous efforts to identify the geological structures in the region and other parts of the basin often involved upward continuation of the potential field data to heights $\:\ge\:500$ m to suppress obscurity caused by cultural features and shallow, non-target sedimentary cover (e.g.,^31,34^). In this study, however, we interpreted the aeromagnetic data directly without upward continuation by computing its derivatives using the β-VDR-with-β-HDR method. In addition to the previous works in the area, we analyzed the magnetic profile across the major geological structures in the area, and estimated the depths to geological structures in 2D and 3D. For both 2D and 3D depth analysis, we assumed a contact or large throw fault model, which corresponds to a structural index of zero (SI = 0). Although the model may not be strictly appropriate for all sources in the area, the depth estimates provide a good set of minimum depths^22^. At locations where there is geological evidence of dikes or sills (which typically have an SI of 1), the calculated depths presented in this study may be multiplied by a correction factor ($\:\sqrt{2}$ according to Eq. 32).

**Fig. 26:**
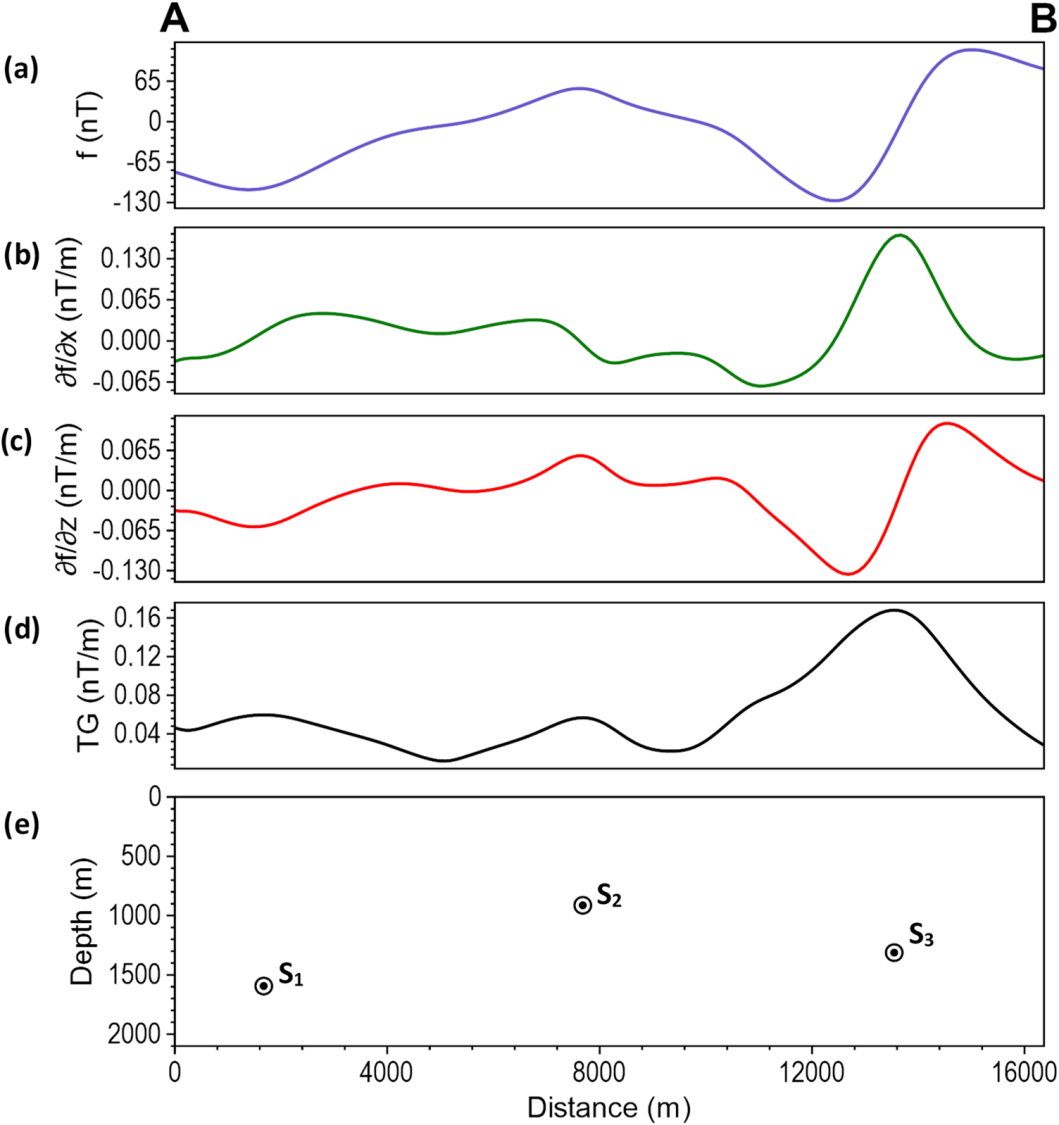
Magnetic Profile Analysis. The (**a**) RTE magnetic anomaly along the profile. (**b**) horizontal derivative (β-HDR) of the anomaly (**c**) vertical derivative (β-VDR) of the anomaly (**d**) TG computed using β-VDR-with-β-HDR method (**e**) locations and estimated depths of subsurface geological structures across the profile.

Figure 25b shows the reduced-to-equator (RTE) map of the study area, which serves as a low-latitude alternative to reduction to pole for providing anomalies that are more symmetrically aligned with their sources. A profile was taken from point ‘A’ to ‘B’ across the magnetic signatures of approximate 2D structures in the area. Figure 26 shows the results obtained from the profile analysis. Figure 26a displays the magnetic anomaly along the profile, while Fig. 26b–d present its β-HDR derivative, β-VDR derivative, and the derived TG, respectively. Three sources labelled S_1_ to S_3_ were obtained from the TG depth analysis, with no spurious solution, as will be demonstrated by their continuity across the profile in 3D analysis. The sources (S_1_, S_2_ and S_3_) are located at approximately 1.67, 7.68 and 13.55 km from the start of the profile and at depths of 1.59, 0.91 and 1.3 km respectively.

**Fig. 27:**
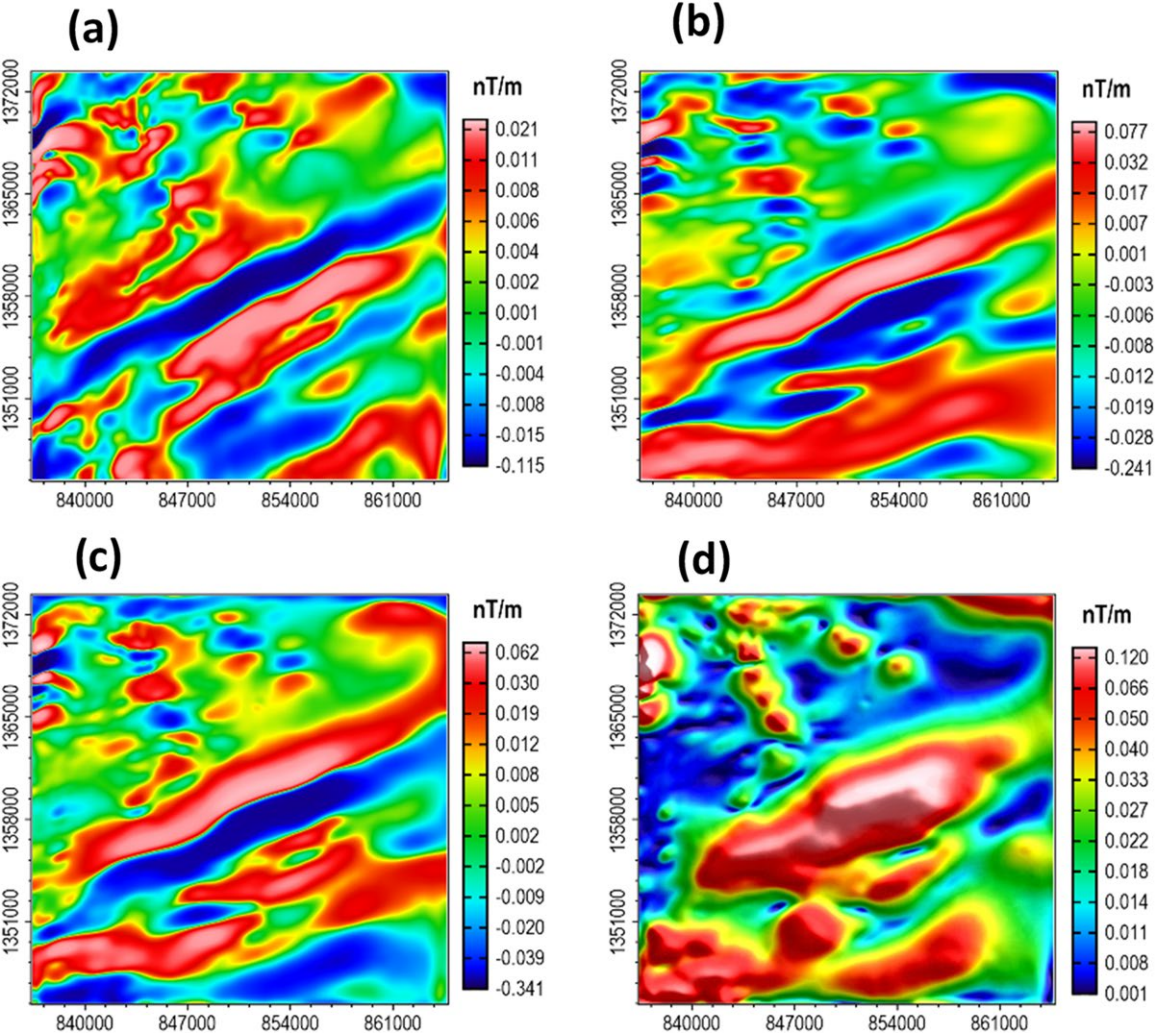
(**a**) β-HDR (x-derivative) (**b**) β-HDR (y-derivative) (**c**) β-VDR (**d**) TG of the magnetic anomaly shown in Fig. 25b.

**Fig. 28:**
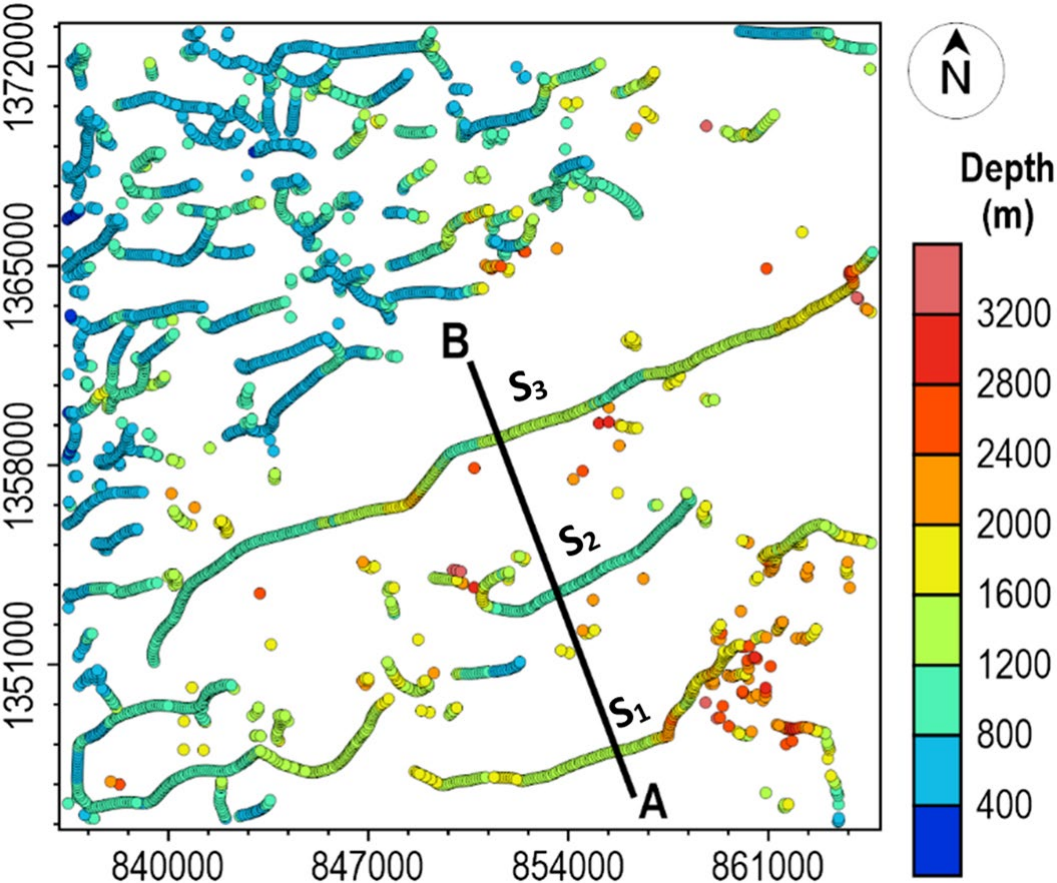
Locations and depths of buried structural features in the study area.

The calculated β-HDR x-derivative, β-HDR y-derivative, β-VDR vertical derivative, and the derived TG from the magnetic grid (Fig. 25b) are presented in Fig. 27a–d, respectively. The 3D solutions (Fig. 28) obtained from the analysis of the TG showed the locations and depths to the top of magnetically discernible geological structures in the area. As demonstrated in our synthetic tests, reliable solutions are closely aligned and form well-defined ridges along linear features, whereas spurious solutions are usually scattered or isolated. According to Phillips et al.^22^, good solutions form well-defined ridges along portions of faults and other linear features. The solutions obtained in this study are well clustered and form well-defined ridges, with a very few isolations, indicating the low noise sensitivity of the β-VDR and β-HDR derivative operators. It should also be noted that volcanic centers (e.g., vertical pipes or irregular intrusions) may produce non-ridge-like TG peaks, and thus, should not be misconceived as spurious solutions.

The clustered solutions revealed that the geological structures in the area dominantly trend ENE-WSW and NE-SW. This aligns well with the regional Pan-African trend of the Nigerian Basement Complex and the dominant trends within the Nigerian Sector of Chad Basin. The study area has a complex stress history as revealed by the presence of significant NW-SE, WNW-ESE and E-W structural trends in the area. The NW-SE and WNW-ESE trends likely represent conjugate shear fractures or localized extensional features formed in response to the main NE-SW and ENE-WSW Pan-African compression or subsequent Mesozoic rifting events. The dominant structural features are interpreted as basement-rooted faults or dike-filled sedimentary fracture systems whose faulting might have resulted from basement fault propagation into the overlying sedimentary column. Evidence of basement fault propagation in the Nigerian sector of the Chad Basin was presented by^29,31^.

The 3D solutions (Fig. 28) showed that the northwestern portion of the study area is dominated by shallow-seated structures whose top depths range between 400 and 800 m. These shallow deformations likely reflect late-stage, near-surface faulting or compaction over buried basement irregularities. Other parts of the study area showed more elongated linear features at deeper depths. The spatial continuity (in the strike direction) of the three identified structures (S_1_, S_2_ and S_3_) from profile analysis is clearly illustrated in the 3D solution map, which also agrees with the 2D results. Generally, the geological structure S_3_ is deeper than S_2_ but shallower than S_1_. At the profile location in the 3D solution map, the depths to S_1_ and S_3_ are classified within the range of 1.2 and 1.6 km, while that of S_2_ is within the range of 0.8 and 1.2 km. This clearly shows quantitative agreement with the 1.59, 0.91 and 1.3 km depth estimates obtained from profile analysis. The results of this real-data application provide a reconnaissance structural model for future exploration, delineating the spatial extent and depth of key geological structures that may control fluid migration pathways and structural traps in the basin.

## Conclusion

We have compacted the β-VDR formula and generalized the method for horizontal derivative calculation to reduce its theoretical computational cost and improve its robustness for edge detection and depth estimation from profile and gridded potential field data.

Our 2D and 3D synthetic tests showed that the original β-VDR method, while being superior to FFT and ISVD, succumbs to the weakness of the FD horizontal derivative when combined for edge detection and depth estimation at high noise levels. This combination resulted in multiple false edge detections and significant underestimation of depths, highlighting a critical failure in combining a well-stabilized vertical derivative with a less-stabilized horizontal derivative. The β-VDR-with-β-HDR method, derived in this study, provides a balanced robustness across all derivative components and maintains exceptional stability across different noise levels. Its TG profile remains smooth, consistently peaks directly over the source edges, and provides reliable depth estimates with minimal spurious solutions even at high noise levels where others failed.

When applied to real data, the β-VDR-with-β-HDR method showed consistency across 2D and 3D datasets and produced results that aligned with the previous knowledge of the area without the conventional practice of initial data filtering.

Considering the superior performance and balanced robustness introduced by the β-HDR to the original β-VDR method, we propose the β-VDR-with-β-HDR method for more robust edge detection and depth estimation from potential field data.

## Data Availability

The generated synthetic data are available from the corresponding author on reasonable request. The aeromagnetic data used in this study are available from the Nigerian Geological Survey Agency (NGSA, https://ngsa.gov.ng/) but restrictions apply to the availability of these data, which were used under license for the current study, and so are not publicly available. Data are, however, available from the authors upon reasonable request and with permission of the NGSA.
